# Multicellular Bacteria Deploy the Type VI Secretion System to Preemptively Strike Neighboring Cells

**DOI:** 10.1371/journal.ppat.1003608

**Published:** 2013-09-05

**Authors:** Christopher J. Alteri, Stephanie D. Himpsl, Shannon R. Pickens, Jonathon R. Lindner, Jonathan S. Zora, Jessa E. Miller, Peter D. Arno, Samuel W. Straight, Harry L. T. Mobley

**Affiliations:** Department of Microbiology and Immunology, University of Michigan Medical School, Ann Arbor, Michigan, United States of America; Osaka University, Japan

## Abstract

The Type VI Secretion System (T6SS) functions in bacteria as a contractile nanomachine that punctures and delivers lethal effectors to a target cell. Virtually nothing is known about the lifestyle or physiology that dictates when bacteria normally produce their T6SS, which prevents a clear understanding of how bacteria benefit from its action in their natural habitat. *Proteus mirabilis* undergoes a characteristic developmental process to coordinate a multicellular swarming behavior and will discriminate itself from another *Proteus* isolate during swarming, resulting in a visible boundary termed a Dienes line. Using transposon mutagenesis, we discovered that this recognition phenomenon requires the lethal action of the T6SS. All mutants identified in the genetic screen had insertions within a single 33.5-kb region that encodes a T6SS and cognate Hcp-VrgG-linked effectors. The identified T6SS and primary effector operons were characterized by killing assays, by construction of additional mutants, by complementation, and by examining the activity of the type VI secretion system in real-time using live-cell microscopy on opposing swarms. We show that lethal T6SS-dependent activity occurs when a dominant strain infiltrates deeply beyond the boundary of the two swarms. Using this multicellular model, we found that social recognition in bacteria, underlying killing, and immunity to killing all require cell-cell contact, can be assigned to specific genes, and are dependent on the T6SS. The ability to survive a lethal T6SS attack equates to “recognition”. In contrast to the current model of T6SS being an offensive or defensive weapon our findings support a preemptive mechanism by which an entire population indiscriminately uses the T6SS for contact-dependent delivery of effectors during its cooperative mode of growth.

## Author Summary

Bacterial Type VI Secretion Systems (T6SS) function as contractile nanomachines to puncture target cells and deliver lethal effectors. Little is known about the lifestyle or physiology dictating when bacteria normally express their T6SS. *Proteus mirabilis* undergoes a characteristic developmental process to coordinate multicellular swarming behavior and discriminates itself from other *Proteus* isolates during swarming, by a visible boundary termed a Dienes line. We report this phenomenon, first noted in 1946, requires the lethal action of the T6SS, T6SS-dependent effectors, and immunity proteins. T6SS-mediated lethality is unique to morphologically distinct swarmer cells, and when it occurs requires direct contact. Using this multicellular system, we report that the pre-formed T6SS strikes neighboring cells upon cell-cell contact. Our findings support a preemptive mechanism by which an entire population indiscriminately uses the T6SS during a cooperative behavior and that social recognition in bacteria is immunity to lethal T6SS attack.

## Introduction

The type VI secretion system (T6SS), a recently discovered Gram-negative secretion pathway [1], [2], delivers effectors upon direct contact with a target cell [3], [4] through a contractile puncturing device. Death of the target cell is the primary outcome that follows the delivery of the lethal effectors, which are translocated from the attacker cell cytoplasm into the periplasm of the target cell via a T6S apparatus in a contact-dependent process [5], [6]. It is well known that bacteria release bactericidal agents such as bacteriocins and antibiotics into the extracellular environment as a means to effectively eliminate bacterial competitors [7]. However, many bacteria survive and replicate in their natural habitat using a multicellular life-style that requires direct contact and cooperativity. As a consequence, we would expect bacteria that have developed multicellular behavior to benefit from a mechanism that depends on cell-cell contact to discriminate between one another and eliminate non-self bacteria, potential cheaters, or competitors from the population. In this regard, T6SS activity, like multicellular behavior, requires direct cell-cell contact. Hence, lethal action of the T6SS would provide a specific advantage for bacteria to discriminate, recognize, and kill competitors that would otherwise interfere or benefit from a cooperative behavior. Surprisingly, despite the strict requirement for cell-cell contact to achieve multicellular behavior and direct contact being required to trigger the contractile T6SS, any relationship between the two remains an open question [8].

*Proteus mirabilis*, a Gram-negative bacterium, undergoes a characteristic developmental process and associated multicellular behavior known as swarming. This unique morphological cycle, differentiating from short (2 µm in length), rod-like vegetative swimmer cells into hyperflagellated, elongated (40 µm in length) swarmer cells, allows *Proteus spp.* to swarm rapidly and uniformly in multicellular rafts across a surface, resulting in a bull's-eye pattern on an agar surface. In 1946, Louis Dienes observed that actively swarming strains of *Proteus* have the remarkable ability to form a visible, macroscopic boundary or “Dienes line” at the intersection of bacterial swarms [9], [10]. Formation of the Dienes line occurs only between swarms of different *Proteus* isolates, demonstrating the ability of one strain of *Proteus* to distinguish itself from another [9], [10]. More than 200 Dienes types have been reported, a diversity that rivals O-serotypes [11]. Although the Dienes phenomenon has been a practical tool in diagnostic settings to type clinical isolates [12], [13], only limited information has been obtained regarding the biology dictating Dienes line formation with the important exception that it has been shown to be dependent on direct cell-cell contact [14].

A recent study [15] identified a six-gene operon in *P. mirabilis* BB2000, termed identity of self or the *ids* genes, and proposed that these genes encode the molecular determinants of self-recognition involved in the Dienes phenomenon. Five of the six *ids* genes were suggested to mediate self-recognition by an unknown diffusible signal [16], [17]. While the mechanism that governs strain recognition and Dienes line formation in *Proteus* remains elusive, it was speculated that lack of self-recognition does not result from killing [15], [16]. While these mechanisms are not understood, the largely descriptive studies on social recognition in *Proteus* uniformly agree that the Dienes recognition phenomenon occurs only between cells that have undergone the morphological and developmental transformation from planktonic cell to highly differentiated swarm cells that are exhibiting multicellular cooperative behavior [18], [19]. Whether in broth or on a surface non-permissive for swarming, undifferentiated *Proteus* that are capable of, but are not actively exhibiting, cooperative behavior, do not discriminate themselves from other strains or demonstrate social recognition [9], [10], [14].

Using a genetic screen, we discovered that the Dienes recognition phenomenon, first noted more than 65 years ago, requires the action of the T6SS. Here, we demonstrate that the presence of a Dienes line correlates with bacterial killing showing that one *P. mirabilis* strain will selectively kill a different *P. mirabilis* strain. A functional T6SS as well as one specific Hcp-VgrG effector-encoding operon among five such operons are required for killing. Thus, our findings elucidate the mechanism that explains social recognition and Dienes line formation. We show that “recognition” is the ability to survive attack from an opposing cell's T6SS. We further investigated the T6SS using our multicellular model system applying the exceptional tools recently developed for studying the T6SS in planktonic cells [20], [21], [22] because the biochemical and genetic studies that have described how bacteria use the T6SS were generally performed under conditions that do not favor natural expression of the specialized secretion system [5], [6], [20], [21], [22], [23], [24]. In contrast to the main conclusion drawn from studying planktonic bacteria, that the T6SS is used for interbacterial competition by functioning solely as either an offensive or defensive weapon [20], our findings provide compelling evidence for a contrarian model to describe how the T6SS is deployed under physiologically relevant conditions during a phase in which differentiated *Proteus* cells interact with one another to swarm. We propose a preemptive antagonism model for T6S that adequately describes contact-dependent delivery of lethal effectors in the context of a natural developmental process that coordinates multicellular behavior.

## Results

### Mutations that affect Dienes line formation map to divergent loci that encode a complete T6SS and Hcp-VgrG effectors

**Figure 1:**
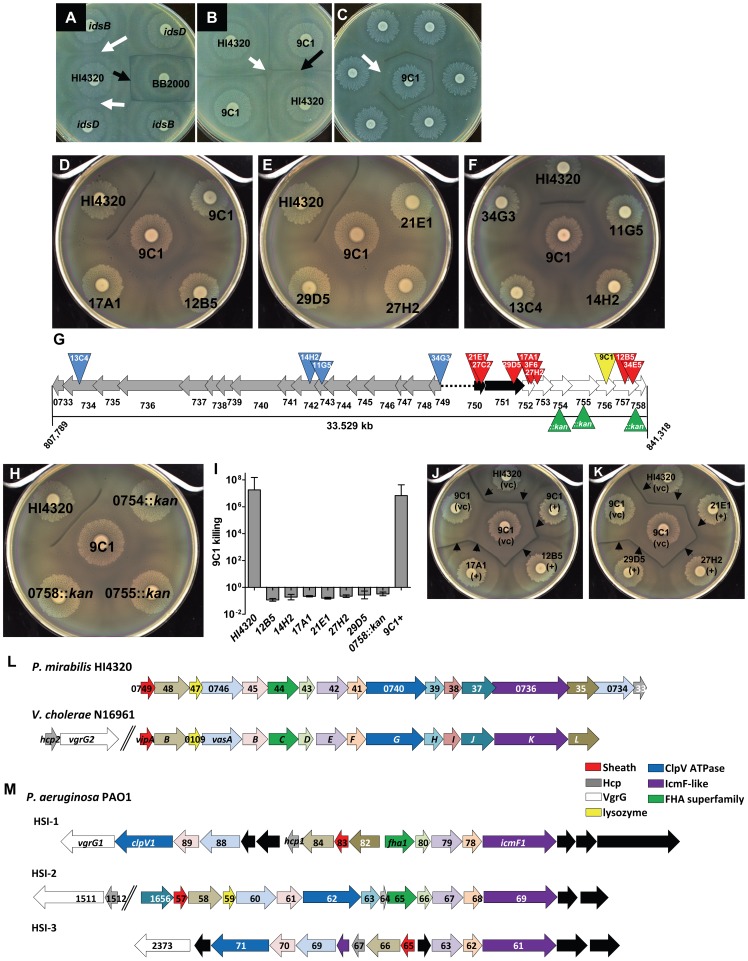
Social recognition and Dienes line formation results from bacterial killing, dependent on the T6SS and divergent *hcp-vgrG* effector operon. (**A**) Non-identical *P. mirabilis* strains, BB2000 and HI4320, form a Dienes line (black arrow). Mutations within *idsB* and *idsD* of HI4320 do not form a Dienes line with their parental strain (white arrows). (**B**) One of 1,920 HI4320 Tn*5* insertion mutants, identified as 9C1, formed a Dienes line (black arrow) with its parental strain. HI4320 merges with itself at the center of the plate (white arrow). (**C**) Transposon mutants were rescreened against 9C1 to find mutants that lost the ability to form a Dienes line (arrow). (**D, E**) Mutant 9C1 merged with itself and insertion mutants 12B5, 17A1, 21E1, 27H2, and 29D5. (**F**) 9C1 merges with T6SS mutants 11G5, 14H2, 13C4, and 34G3. (**G**) Transposon insertions (red triangles) in genes located within the same operon as the 9C1 mutant (yellow triangle). The three genes not identified in the genetic screen were disrupted by targeted insertion of antibiotic resistance gene (green triangles). *hcp* and *vgrG* homologs (black arrows) and additional genes predicted to encode effectors delivered by the T6SS (white arrows) are indicated. Additional mutants (13C4, 14H2, 11G5, and 34G3) have transposon insertions (blue triangles) in genes encoding conserved components of the T6SS (grey arrows). The black dotted line represents intergenic sequence. (**H**) Mutants PMI0754*::kan*, 0755*::kan*, and 0758*::kan* merge with 9C1. (**I**) Killing assays confirming 9C1 killing are reported as [(CFU of test strain/CFU of 9C1)_output_]/[(CFU of test strain/CFU of 9C1)_input_]. Strain HI4230 kills mutant 9C1 by at least 7-logs. Mutant 9C1 complemented with the primary *hcp-vgrG* effector operon (9C1+) kills mutant 9C1. (**J, K**) Dienes line formation (arrows) was restored when the indicated mutants are complemented with the entire primary *hcp-vgrG* effector operon, (+); empty vector, (vc). (**L**) The 17 genes that encode the HI4320 T6SS are highly conserved with the well characterized T6SS genes from *V. cholerae* N16961 and have identical gene order in their respective chromosome and (**M**) comparison to the three known T6SSs (HSI-1, HSI-2, and HSI-3) encoded within the genome of *P. aeruginosa* PA01. The arrows are color-coded based upon known or predicted gene function. Black arrows represent genes that are not found in either *P. mirabilis* HI4320 or *V. cholerae* N16961 T6SS gene locus. See also Table S1.

It has been recently proposed that the *ids* genes of *P. mirabilis* are sufficient for self-recognition and Dienes line formation [16]. However, as a part of this study, we report that *ids* mutants constructed in the well characterized prototype strain HI4230 have no defect in self or non-self identity (Fig. 1A, white arrows). Non-identical strains, HI4320 and BB2000, form a Dienes line (Fig. 1A, black arrow), while identical strains of HI4320 merge (Fig. 1B, white arrow). We used an approach similar to the one used to identify *ids* in BB2000 [15], by first identifying a single gene in strain HI4320 involved in the Dienes phenomenon using a transposon screen of *P. mirabilis* HI4320. Only one of 1920 insertion mutants, identified as 9C1, formed a Dienes line with its wild-type parental strain HI4320 on swarm agar (Fig. 1B, black arrow).

Using our genetic approach, however, we identified additional genes required for the Dienes phenomenon by re-screening the insertion mutants for those that lost the ability to form a Dienes line with mutant 9C1 (Fig. 1C, white arrow). Twelve mutants merged with 9C1, suggesting a role in strain recognition (Fig. 1D–F). Remarkably, 8 of 12 mutants have transposon insertions in genes localized to the same operon (Fig. 1G, red triangles) as the original 9C1 mutant (Fig. 1G, yellow triangle). The four additional mutants that merged with 9C1 (Fig. 1F) have transposon insertions in four of the 17 contiguous genes (Fig. 1G, blue triangles) that encode highly conserved structural components of the T6SS (Table S1) [25]. These genes reside on an adjacent but divergent gene cluster beginning with PMI0749 (COG3516), which encodes a homolog of the *Vibrio cholerae* VipA T6SS sheath protein (Table S1).

PMI0750 and PMI0751 belong to the *hcp* (COG3157) and *vgrG* (COG3501) superfamilies, respectively, and encode proteins that could assemble to form the puncturing device of the T6SS [1]. Located directly downstream and within the same potential operon as PMI0750 and PMI0751 are genes PMI0752–PMI0758. We predict that these genes encode T6SS-dependent effectors because 9 transposon mutants within this putative operon were identified that affected either recognition or killing. Furthermore, we predicted that PMI0754, PMI0755, and PMI0758, which were not identified in the screen, also play a role in T6S-dependent recognition or killing. Indeed, strains with mutations in each of these three genes (Fig. 1G, green triangles), constructed in strain HI4320; 0754*::kan*, 0755*::kan*, and 0758*::kan*, merged with 9C1 (Fig. 1H). Thus, independent disruption of every gene within the identified operon by either transposon insertion or by targeted knock-out abolishes Dienes line formation with the susceptible 9C1 mutant suggesting that the entire *hcp-vgrG* effector operon, encoded by PMI0750–PMI0758, is required for complete function.

### Dienes line formation is associated with bacterial killing during multicellular behavior

As will be discussed, the observation that the entire *hcp-vgrG* effector operon is required for Dienes line formation and that recognition ultimately arises from the reaction occurring when two bacterial cells use the T6SS to deliver effectors to one another suggests directionality; two strains attempting to kill one-another. To explore this possibility, we first sought to understand whether the Dienes recognition phenomenon results from killing. We hypothesized that bacteria in the area of the Dienes line would be permeable to the fluorescent DNA stain SYTO 9 due to the loss of membrane integrity and viability resulting from the lethal action of the opposing swarm's T6SS. Indeed, bacteria in the Dienes demarcation can be brightly stained by the vital dye (Fig. S1), which indicates a loss of viability and killing, occurs at the boundary between swarms.

To quantify killing, competition assays were conducted between *P. mirabilis* HI4320 and mutant 9C1. Wild-type HI4320 killed mutant 9C1 by at least 7-logs when plated together on agar to permit swarming and co-cultured for 18 h (Fig. 1I). Further, we find 10^6^ fewer 9C1 than were co-inoculated with HI4320, which must be explained by killing. Insertion mutants of the *hcp-vgrG* effector operon and the T6SS merged with mutant 9C1 and were unable to kill 9C1 (Fig. 1D–F; Table 1). Dienes line formation was restored when mutants of the *hcp-vgrG* effector operon were complemented with a plasmid carrying the entire *hcp-vgrG* effector locus (Fig. 1J and 1K). As expected, mutant 9C1, when complemented with the *hcp-vgrG* effector operon (9C1+), reverted back to a wild-type Dienes phenotype and was able to kill 9C1 (Fig. 1I and 1J). Complementation analysis confirms that the *hcp-vgrG* operon encodes factors required for Dienes line formation and for killing or for immunity to killing (*i.e.*, recognition). The remarkable conservation between the newly identified *P. mirabilis* T6SS and the best-characterized T6SSs from *V. cholerae* (Fig. 1L) and *P. aeruginosa* (Fig. 1M) strongly suggests that the T6SS could function to deliver lethal effectors to target cells during interbacterial competition in *P. mirabilis*.

**Table 1:** The T6SS and primary effector operon are required for killing.

	Mean Input CFU		Mature Swarm CFU	
	Antagonist	9C1	Antagonist	9C1
**HI4320**	1.82×10^7^	3.89×10^7^	7.06×10^11^	**1.00×10^2^**
**12B5**	3.33×10^7^	2.52×10^7^	1.85×10^10^	1.83×10^9^
**14H2**	2.92×10^7^	2.54×10^7^	2.27×10^10^	2.45×10^9^
**17A1**	2.90×10^7^	3.03×10^7^	1.76×10^10^	3.41×10^9^
**21E1**	3.29×10^7^	3.03×10^7^	2.43×10^10^	2.43×10^9^
**27H2**	2.64×10^7^	2.82×10^7^	2.23×10^10^	4.23×10^9^
**29D5**	3.04×10^7^	2.65×10^7^	1.69×10^10^	2.07×10^9^
**9C1+**	1.82×10^7^	2.92×10^7^	1.79×10^10^	**1.00×10^2^**

### Visualization of T6S-dependent cell-cell interactions during multicellular swarming

Despite killing of one strain by another, the macroscopic Dienes line is fixed at the midpoint between swarms. To observe the events immediately following the merger of two swarms, where it is expected that direct contact and T6S-dependent interactions occur, we performed live-cell microscopy. Cultures of *P. mirabilis* HI4230 producing green fluorescent protein (GFP) and cultures of mutant 9C1 producing dsRed were spotted on opposite sides of a swarm agar plate and were observed in real-time as the bacterial strains swarmed toward one another (Movie S1). The strains merge and seamlessly interdigitate as cells from opposing swarms first come into direct cell-cell contact (Movie S1).

**Figure 2:**
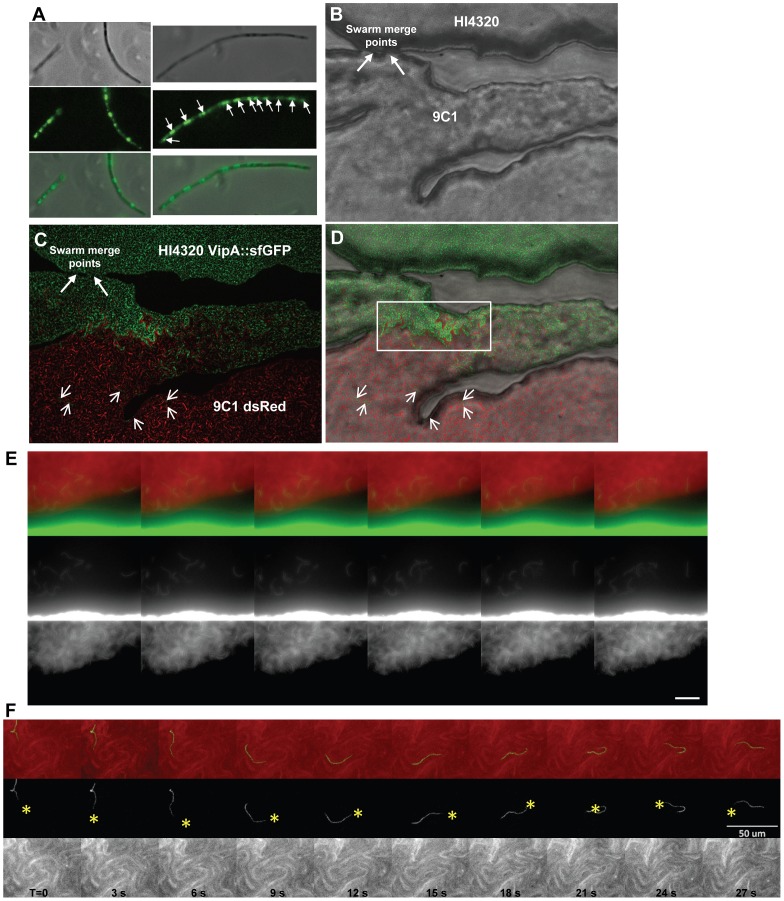
Visualization of the T6SS activity during multicellular infiltration and killing. (**A**) Actively swarming *P. mirabilis* HI4230 cells expressing VipA::sfGFP viewed under phase contrast and by fluorescence microscopy. Punctate green staining represents sites of T6SS assembly (arrows). (**B**) HI4320 and mutant 9C1 are observed merging at their swarm fronts on an agar surface (arrows) under phase contrast. (**C**) HI4320 VipA::sfGFP is observed infiltrating deep into the opposing 9C1 swarm expressing dsRED. Short arrows indicate individual HI4320 swarm cells within the 9C1 swarm. (**D**) Merged image of (B) and (C). The boxed region encapsulates intense green straining representing increased sfGFP-signal due to increased assembly of the T6SS sheath (VipA::sfGFP) by the front swarm edge of wild-type HI4320. (**E**) Visualization of individual HI4320 wild-type swarmer cells (green) that have infiltrated and are demonstrating multicellular swarming within the susceptible 9C1 swarm (red). (**F**) The forward movement of one individual HI4320 swarm cell over 30 seconds is indicated by yellow asterisk. Elapsed time (T) in seconds is indicated. Each frame in (E, F) represents 3 seconds elapsed time and the white bar is 50 µM. See also Figure S1, S2, and Movie S1 and S2.

To obtain greater resolution and specificity of the lethal T6S-dependent interaction and visualize the aggressor during associated killing, we constructed a strain containing a fusion between the gene encoding HI4230 VipA (PMI0749) and the gene for super folder green fluorescent protein (sfGFP) in a fashion similar to methods used in *V. cholerae* studies [22]. Seminal work by the Mekalanos Laboratory has rapidly extended understanding of the biomechanics and timing for assembly, disassembly, and contraction of the T6SS nanomachine in both *V. cholerae* and *P. aeruginosa* [1], [2], [20], [22]. These pioneering studies have validated a number of tools that can be applied to any T6SS of interest, given the high degree of conservation of core T6SS genes across a wide-range of bacterial taxa. Specifically, it is now established that cycles of T6SS assembly and contraction, or T6SS activity, can be directly visualized using fluorescent protein fusions to orthologs of the *V. cholerae* T6SS sheath protein VipA [20]. Fusion of sfGFP to the T6SS sheath protein (VipA) encoded by PMI0749 causes a punctate pattern of fluorescence emitted from sfGFP (Fig. S2) in HI4320 during swarming and infiltration of 9C1 expressing dsRED (Fig. S2). This is in contrast to the diffuse fluorescence emitted when sfGFP is independently expressed (Fig. S2). Actively swarming HI4230 cells expressing VipA::sfGFP were removed from the agar plate and individual swarmer cells were observed to emit punctate green staining over the length of the cell (Fig. 2A). The green staining was not diffuse throughout the swarm cell nor observed at only one location (Fig. 2A), suggesting that VipA is localized in discrete regions along the length of the elongated cell and the T6SS is assembled at numerous sites (Fig. 2A). In contrast to *V. cholerae* (2–3 µM in length) with a single site of T6SS assembly [20], [22], the much longer wild-type swarmer cell of *P. mirabilis* (∼40 µM in length) has many more T6SS assembly sites; approximately 12 areas have intense green staining (Fig. 2A).

Examination of the T6S-dependent interaction between 9C1 expressing dsRed and wild-type HI4230 expressing VipA::sfGFP revealed a one-sided invasion by the dominant HI4320 (Fig. 2B–D). By 10 min post-merge, massive infiltration of 9C1 dsRed by HI4320 VipA::sfGFP (Fig. 2C) was observed. In contrast, 9C1 dsRed swarmer cells were rarely observed on the opposite side of the swarm merge points even at this early time post-merge (Fig. 2D). This could reflect the fact that HI4320 efficiently kills 9C1 when their multicellular swarms come into contact. It is important to note that the individual wild-type HI4320 VipA::sfGFP- expressing swarmer cells (green) located within the 9C1 mutant swarm (red) are not co-localized (yellow) but rather appear aligned (green) directly against mutant 9C1 swarmer cells (red) (Fig. 2C). An apparent increase in VipA::sfGFP signal appears in HI4320 at the vicinity with the greatest degree of contact between mutant 9C1 and advancing wild-type swarm (boxed area, Fig. 2D). This was unexpected because the VipA::sfGFP fusion is induced by arabinose, which is uniformly present (10 mM) throughout the plate. We hypothesize that the physical interaction between opposing swarm populations at the edge of the leading swarm front triggers rapid assembly and disassembly of the multiple VipA subunits that compose the T6SS sheath [22] because it has been previously demonstrated that when a cell expresses a fusion of a fluorescent protein to an orthologous VipA, the intensity of green staining (sfGFP) directly correlates to greater T6SS sheath assembly at the area of inter-strain contact that reflects delivery of the cell-puncturing device, encoded by *hcp* and *vgrG*, and effector proteins [20], [22].

**Figure 3:**
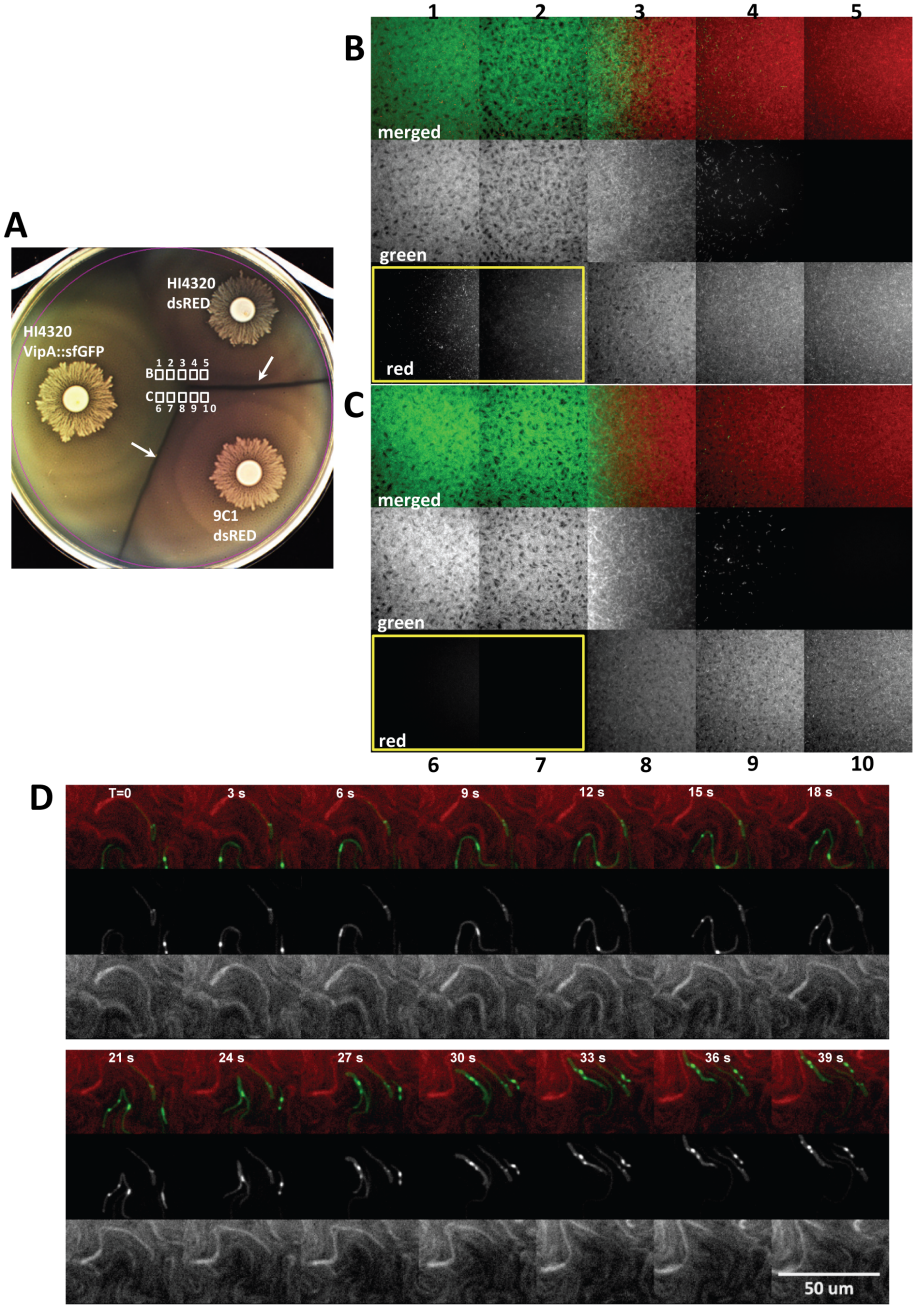
Infiltration of resistant and sensitive opposing swarms by *P. mirabilis* HI4320 expressing VipA::sfGFP. (**A**) Agar plate inoculated with *P. mirabilis* HI4320 VipA::sfGFP opposing strains HI4320 or mutant 9C1 expressing dsRED. Dienes lines (white arrows) formed between HI4320 VipA::sfGFP or HI4320 dsRED and 9C1 dsRED but not between HI4320 VipA::sfGFP and HI4320 dsRED. The fields examined by fluorescence microscopy (white boxes) are indicated at the intersection of the swarms. Active infiltration of (**B**) HI4320 dsRED and (**C**) mutant 9C1 dsRED by HI4320 expressing VipA::sfGFP. The numbered panels in (B) and (C) correspond to the numbered areas indicated in (A) and were viewed directly on the agar plate at the time of intersection. For each field, the individual green and red channels from the merged image are shown to maximize visualization of the infiltrating swarm. In (B) and (C) the panels boxed with a yellow border show dsRED-expressing bacteria infiltrating into the HI4320 VipA::sfGFP swarm are only observable with HI4320 dsRED (B1 and B2 red); the susceptible 9C1 dsRED is undetectable (C6 and C7 red). (**D**) Infiltrating HI4320 expressing VipA::sfGFP demonstrate numerous areas of T6SS activity (green) when in direct contact with target 9C1 cells expressing dsRED. Elapsed time (T) is indicated in seconds. See also Movie S3, S4, S5.

To examine this infiltration in real-time, we imaged the area approximately 3 mm beyond the merge point on the 9C1 dsRed side where there are no observable swarms of multicellular HI4320 expressing VipA::sfGFP (Movie S2). Only individual wild-type HI4320 swarmer cells were observed deep within the 9C1 swarm population and appeared to maintain the ability to migrate rapidly within the opposing swarm (Fig. 2E and 2F) (Movie S2). To test the hypothesis that the lethal activity of the HI4320 T6SS against mutant 9C1 prevents the susceptible strain from infiltrating the wild-type swarm, we examined the intersection between HI4320 VipA::sfGFP and HI4320 expressing dsRED, and HI4320 VipA::sfGFP and 9C1 expressing dsRED, on the same agar plate (Fig. 3A). While numerous dsRED-expressing HI4320 are readily observed penetrating the HI4320 VipA::sfGFP swarm (yellow boxed area, Fig. 3B), it was not possible to detect dsRED-producing 9C1 within the wild-type swarm (yellow boxed area, Fig. 3C). Close examination of the infiltrating cells in real-time reveals single HI4320 swarm cells, producing the T6SS sheath VipA fused to sfGFP, in direct contact with a 9C1 target cell within the infiltrated swarm (Fig. 3D), demonstrating a role for T6S-dependent activity during multicellular behavior.

### Genomic organization of a prototypical T6SS *hcp*-*vgrG* effector operon

**Figure 4:**
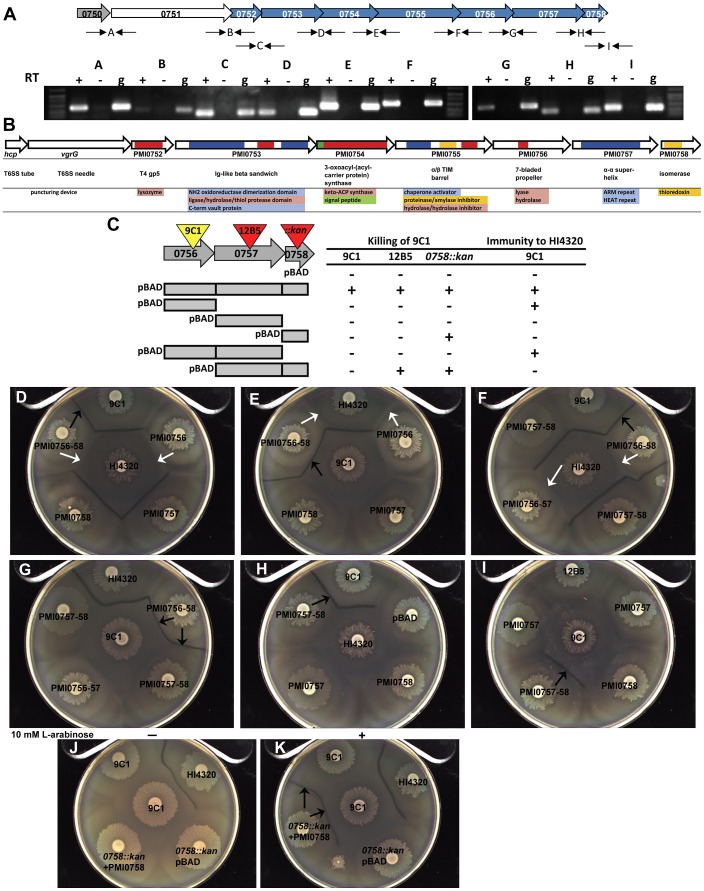
The primary *hcp-vgrG* T6SS effector operon encodes both killing and immunity functions. (**A**) Transcription of the primary effector operon based upon RT-PCR. Intergenic primer pairs (thin black arrows) flanking the open reading frames of PMI0750-PMI0759 are represented by letters. For each reaction (+) cDNA made from RNA using reverse transcriptase, (−) no RT enzyme, or (g) genomic DNA purified from HI4320 were used as template for PCR. (**B**) Predicted structural homology and potential functions for the effectors and other proteins encoded within the primary *hcp-vgrG* operon. Functional domains are color coded beneath each gene. (**C**) Wild-type HI4320, transposon mutants 9C1 and 12B5, and mutant *0758::kan* expressing pBAD empty vector or pBAD containing the indicated genes were inoculated onto agar containing 10 mM L-arabinose opposing mutant 9C1 containing empty vector. The presence of a Dienes line (+) indicates killing of 9C1. Mutant 9C1 expressing the same constructs were also examined against wild-type HI4320 for complementation (+) of the 9C1 immunity defect as indicated by absence of a Dienes line (immunity to HI4320). (**D–G**) *P. mirabilis* HI4320 and mutant 9C1 (center) were assessed for Dienes line formation against 9C1 with pBAD containing the indicated genes. PMI0756 is necessary and sufficient to restore 9C1 immunity against parental HI4320 (white arrows), while all three genes encoded by PMI0756, PMI0757, and PMI0758 are necessary and sufficient to restore both 9C1 immunity against HI4320 and 9C1 killing of 9C1 lacking PMI0756 (black arrows). (**H, I**) Mutant 12B5 (peripheral swarms) contains a transposon insertion in PMI0757 and is unable to kill mutant 9C1 unless 12B5 is complemented with pBAD containing PMI0757 and PMI0758. Parental HI4320 does not form a Dienes line with mutant 12B5 (pBAD). Complementation of 12B5 and the resulting Dienes line formation with 9C1 are indicated with black arrows in (H) and (I). All plates in (D–I) contain 10 mM L-arabinose and swarms labeled HI4320, 9C1, and 12B5 contain pBAD empty vector. (**J**) HI4320 lacking PMI0758 (0758::*kan*) carrying an uninduced clone of PMI0758 on pBAD does not form a Dienes line with mutant 9C1 in the absence of arabinose (−). (**K**) The ability to form a Dienes line with mutant 9C1 is fully restored by arabinose induction of the pBAD promoter (+) to express PMI0758 (*0758::kan* +PMI0758). See also Figure S3 and S4.

To assess operon structure, we performed RT-PCR to identify cDNA representing a transcript that spans the junctions between the ORFs within the putative *hcp*-*vgrG* effector operon (Fig. 4A). It was possible to detect and amplify cDNA representing each junction between all of the predicted ORFs from the identified operon when reverse transcriptase was included (+) in the cDNA synthesis reaction (Fig. 4A). The same products were observed when genomic DNA extracted from HI4320 was used as the template (g); however, it was not possible to amplify the same products when reverse transcriptase was excluded from the cDNA synthesis reaction (−) (Fig. 4A). Thus, the *hcp-vgrG* gene cluster representing genes PMI0750–PMI0758, is co-transcribed as a single operon; however, the possibility that additional promoters are present within the operon cannot be ruled out based on these results.

To gain clues as to how the identified effector operon confers both killing and immune functions, we employed bioinformatic analyses on the predicted proteins encoded by the operon. It is known from studies in other bacteria that Hcp and VgrG, encoded by the first genes within the operon, are structural components of the T6SS that form a hollow tube (Hcp subunits) that could be capped with a puncturing tip (VgrG) [24]. It is inside this hollow tube made from numerous copies of the Hcp monomer, where lethal effectors presumably reside. Despite the remarkable structural similarity between the Hcp-VgrG T6SS effector delivery complex and the bacteriophage T4 gp5–gp27 cell-puncturing device, the possible T6SS puncturing tip, VgrG, lacks a lysozyme domain [24]. The third gene of the primary *hcp*-*vgrG* effector operon, PMI0752, has predicted structural homology to T4 gp5 lysozyme-containing domain (Fig. 4B). Having predicted lysozyme activity encoded in a protein separate from VgrG, could represent a similar strategy used by bacteriophage that lack a canonical lysozyme domain within their orthologous gp5–gp27 puncturing device [24], [26]. Transposon insertion into this putative lysozyme-encoding gene results in the loss of 9C1 killing (Fig. 1D and 1I, 17A1), yet 9C1 is not affected by production of the PMI0752 gene product, even when it contained the HI4320 PhoA signal peptide (Fig. S3). This result suggests that the PMI0752-encoded lysozyme fulfills the T6SS cell wall-puncturing function, being required for delivery of another effector rather than a performing a lethal effector function itself.

While PMI0750–PMI0752 appear to encode three proteins that together might comprise the T6SS puncturing device with lysozyme activity to breach the target cell wall, the remaining proteins encoded by the operon have less obvious relationships to other well-characterized proteins. PMI0753 has a modeled structure with homology to proteins that contain an Ig-like β sandwich fold with thiol protease activity (Fig. 4B). It is likely that PMI0754 encodes a protein that is not delivered into the target cell (*i.e.*, not an effector), because it is predicted to contain a signal peptide and would be secreted into the periplasm of the host bacterium via the general secretory pathway. PMI0754 is also predicted to possess 3-oxoacyl-(acyl carrier protein) synthase activity (Fig. 4B) and could serve to acylate an immunity or effector protein to tether the protein within the periplasm, as has been suggested to occur for immunity proteins in *P. aeruginosa* [6]. PMI0755 encodes a protein of unknown function that contains a predicted α/β TIM barrel with putative structural homology to amylase/proteinase/hydrolase proteins (Fig. 4B) and appears to contain a C-terminal Endo VII/HNH superfamily nuclease domain.

Mutant 9C1 has a transposon insertion in PMI0756 and as a result is susceptible to T6SS-dependent lethal activity delivered by parental wild-type cells. PMI0756 is predicted to have a 7-bladed propeller fold and has a predicted hydrolase domain (Fig. 4B). PMI0757 encodes a predicted α-α superhelical protein that has modeled structural homology to Armadillo (ARM) repeat proteins. It is notable that both 7-bladed propeller proteins and ARM repeat proteins often promote protein-protein interactions in eukaryotic cells [27], [28]. The last gene in the identified *hcp*-*vgrG* effector operon is predicted to be a thioredoxin isomerase (Fig. 4B). In bacteria, thioredoxin activity is generally important for proper inter- or intra-molecular disulphide formation among exported proteins. Since the T6SS delivers proteins from the cytoplasm into the periplasm of a target cell, bypassing the originating cell's own periplasm, the putative thioredoxin encoded by PMI0758 could be important for proper folding of effectors upon access to the target cell periplasm. In support of PMI0758 being required for lethal effector function, mutation of the potential thioredoxin (PMI0758::*kan*) abolishes killing of 9C1 (Fig. 1H).

### A single T6SS-dependent *hcp*-*vgrG* effector operon encodes killing and immunity functions

Since the transposon insertion in PMI0756 (*i.e.*, 9C1) in HI4320 results in 9C1 being susceptible to the lethal action of the wild-type T6SS, it follows that 9C1 has lost an immunity function. However, disruption of PMI0756 also results in the inability of 9C1 to exert lethal action on itself (Fig. 4C, 9C1 pBAD), despite 9C1 being susceptible to T6SS-dependent killing. Complementation of 9C1 with the entire *hcp-vgrG* operon on a plasmid restores both immunity against wild-type and the ability to kill and form a Dienes line with 9C1 containing an empty vector, demonstrating that there are no mutations outside of the *hcp-vgrG* operon that are responsible for the phenotype displayed by the 9C1 mutant (Fig. 1I and 1J). To determine the contribution of PMI0756 to immunity and killing, the PMI0756 gene from wild-type HI4320 was cloned into a plasmid containing an arabinose-inducible promoter and introduced into 9C1. In the absence of arabinose, a Dienes line formed between 9C1 pBAD0756 and wild-type HI4320, while no demarcation was observed between 9C1 pBAD0756 and 9C1 containing an empty vector (data not shown). Arabinose induction of PMI0756 in 9C1, however, restores 9C1 immunity to wild-type HI4320 killing as shown by the absence of a Dienes line (Fig. 4D, white arrows), but does not provide 9C1 with the killing function required to form a Dienes line with 9C1 containing empty vector because a Dienes line is not formed between their respective swarms (Fig. 4E).

That PMI0756 restored 9C1 immunity to wild-type HI4320 but not killing of susceptible 9C1, and insertions downstream of PMI0756 [namely 12B5 (PMI0757::*tn*) and PMI0758::*kan*] lose the ability to kill 9C1 but remain immune to wild-type HI4320, it is likely that the transposon insertion in PMI0756 in 9C1 affects production of one or both of the downstream gene products that are required for killing (Fig. 4C). Indeed, while PMI0756 is necessary and sufficient to restore 9C1 immunity against parental HI4320 (Fig. 4D–F, white arrows), all three genes encoded by PMI0756, PMI0757, and PMI0758 are required to restore both 9C1 immunity against HI4320 and 9C1 killing of 9C1 lacking PMI0756 (Fig. 4D–G, black arrows). In support of a specific role in killing, loss of PMI0757, PMI0757 and PMI0758, or PMI0758 alone, abrogates 9C1 killing by HI4320 (Fig. 4C) because mutant 12B5 can be complemented to form a demarcation with 9C1 only when PMI0757 and PMI0758 are both present (Fig. 4H and 4I). PMI0758::*kan* also retains immunity to wild-type HI4320 (Fig. 4J, *0758::kan* pBAD) but requires induction of PMI0758 to restore killing and Dienes line formation with 9C1 (Fig. 4K, black arrows).

It is notable that insertions within the identified *hcp-vgrG* effector operon, in any gene upstream or downstream of PMI0756, abrogates the ability to kill and form a Dienes line with 9C1 but does not cause loss of immunity to wild-type (Fig. 1D–F) (Fig. S4), suggesting that: 1) each of the gene products from PMI0750 (*hcp*) through PMI0755, in addition to PMI0757 and PMI0758, provide T6SS-dependent killing function; 2) that PMI0756 is required for both immunity and killing; and 3) that PMI0756 is the sole gene product encoded by this *hcp*-*vgrG* locus that is necessary for immunity against the lethal T6SS-dependent activity of the putative effector operon.

### Lack of T6SS-dependent killing allows non-identical wild-type swarms to merge

**Figure 5:**
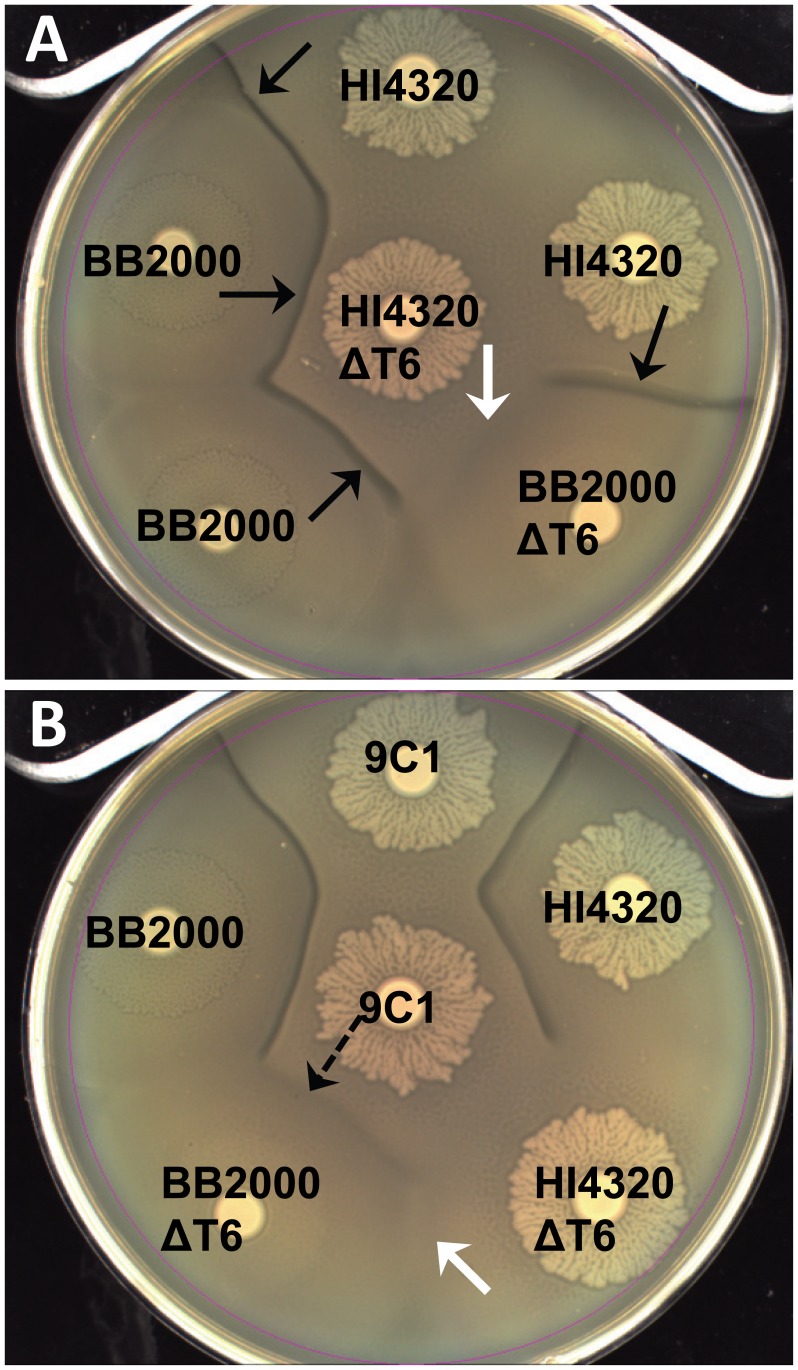
Contact-dependent preemptive antagonism is dependent on the T6SS. (**A**) A Dienes line (black arrows) forms between two different wild-type isolates, HI4320 and BB2000 (strain A and B kill each other). Loss of the T6SS (ΔT6) in either isolate by disruption of PMI0742 does not affect the discriminatory Dienes line (strain A kills strain B or strain B kills strain A). Loss of the T6SS in both isolates allows non-identical swarms to merge and the lack of T6SS-dependent killing appears as recognition (white arrow). (**B**) Mutant 9C1 maintains the ability to form a Dienes line with non-identical BB2000 lacking a T6SS (dashed arrow). No line appears when HI4320 and BB2000 both lack the T6SS (white arrow). See also Figure S5.

While it was possible to isolate mutants unable to kill an immune-deficient mutant (9C1) and thus, identify T6S-dependent effectors, it is not possible to understand the biology that dictates use of T6S in a natural system since once immunity is restored, the deficient strain will not kill the parent strain, as they are otherwise isogenic. We presume the natural Dienes phenomenon, which we have now shown requires the lethal action of the T6SS, could result from both strains killing each other (*i.e.*, preemptive antagonism) following the initial signal of direct cell-cell contact. To determine if the interaction observed between wild-type swarms depends on the activity of the T6SS, we disrupted the T6SS (Fig. S5) in another *P. mirabilis* isolate, strain BB2000 (BB2000 ΔT6) using allelic replacement. When strain HI4320 and strain BB2000 swarms meet, a Dienes line is visible (Fig. 5A) since by definition the Dienes line forms between non-identical multicellular swarms [9], [10]. Interestingly, when one of the opposing strains, either BB2000 or HI4320 is missing the action of the T6SS (BB2000ΔT6 or HI4320ΔT6), the Dienes line is indistinguishable from the one that forms between HI4320 and BB2000 wild type strains that are both competent for T6S-dependent killing (Fig. 5A, black arrows). However, when both HI4320 and BB2000 lack their respective T6SS (BB2000ΔT6 and HI4320ΔT6), the demarcation line does not form and the non-identical swarms merge in a manner identical to HI4320 or BB2000 merging with themselves (Fig. 5A and 5B, white arrows). Thus, the experiments confirm that killing is T6-dependent and swarm cells of both strains are capable of killing each other following direct contact. Even the 9C1 mutant, which is unable to attack using the primary effector operon, appears to use the T6SS with another effector since it makes a demarcation with BB2000 lacking a functioning T6SS (Fig. 5B, dashed arrow). That 9C1 is capable of forming a Dienes line with the T6SS-deficient BB2000 supports indiscriminate firing of the T6SS during swarming and that 9C1 is resistant to self-killing, both are necessary for a preemptive antagonism model for the T6SS.

### Multiple *hcp-vgrG* effector operons are expressed during *P. mirabilis* HI4320 swarming

**Figure 6:**
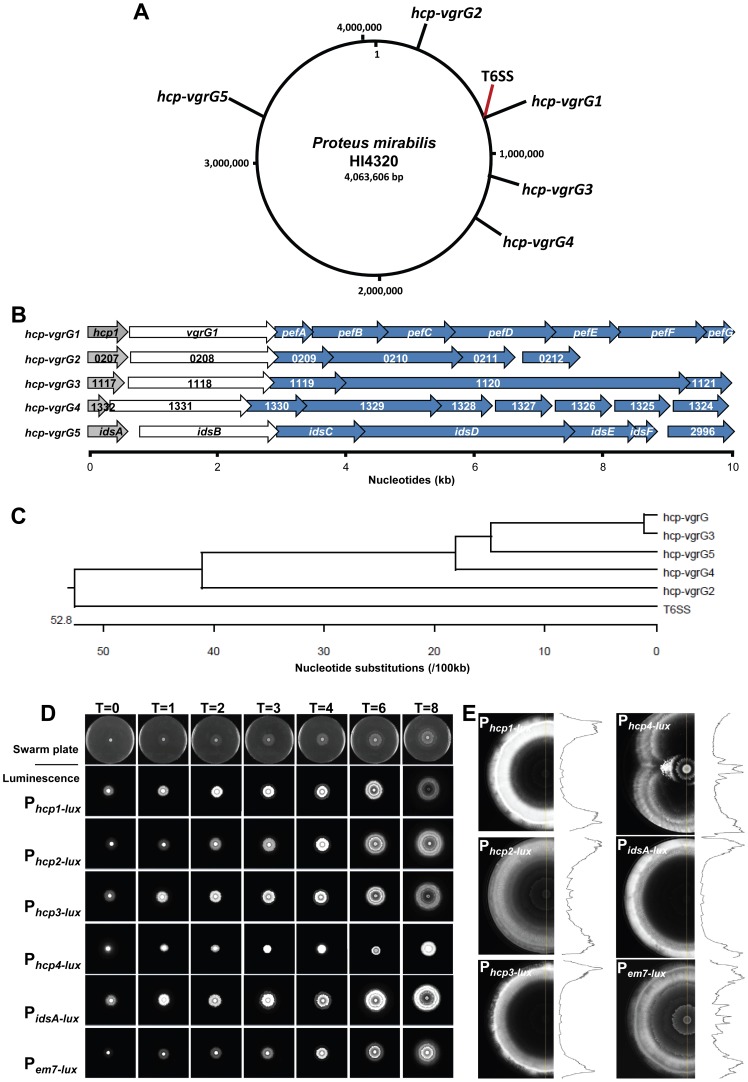
The *P. mirabilis* HI4320 genome contains one primary and four orphan *hcp-vgrG* effector operons that are expressed during swarming. (**A**) A circular representation of the *P. mirabils* HI4320 genome depicting the location of the primary *hcp-vgrG* effector operon (*hcp-vgrG1*), divergent T6SS, and the four orphan *hcp-vgrG* effector operons (*hcp-vgrG2-5*). (**B**) PMI0750–PMI0758 encode the primary *hcp-vgrG1* effector operon (*pef*) adjacent to the T6SS operon (see Figure 2); PMI0207–PMI0212 encode the *hcp-vgrG2* effector operon; PMI1117–PMI1121 encode the *hcp-vgrG3* effector operon; PMI1332-PMI1324 encode the *hcp-vgrG4* effector operon; and PMI2990–PMI2996 is the *ids* operon (*hcp-vgrG5*). Genes with homology to *hcp* (grey), *vgrG* (white), and predicted T6SS effectors (blue) are shown. (**C**) Alignment of nucleotide sequences of the putative promoter regions upstream of the *hcp* genes and PMI0749 (*vipA*) the first gene of the T6SS operon. (**D**) *P. mirabilis* HI4320 expressing *hcp* promoter::*luxCDABE* transcription fusions were observed over time during active swarming. Individual promoter fusions are indicated to the left of the swarm panels and the time (T) in h is indicated. A representative time course of swarming is shown in the top row for reference. (**E**) A separate agar plate was inoculated to capture the 18 h time point before the swarm front reached the edge of the plate for each *hcp* promoter-luciferase transcriptional fusion. Density traces (black line) are shown to visualize changes in luminescence throughout the entire multicellular swarm population. See also Figure S6, S7, S8, S9.

Due to the discrete organization of the identified effector genes linked to genes that encode Hcp and VgrG structural components (Fig. 4B) into a single transcriptional unit (Fig. 4A) distinct from the conserved T6SS gene cluster (Fig. 1G), we sought to determine if additional orphan effector operons could be identified by scrutinizing the genome for *hcp/vgrG* gene pairs. The prototype *P. mirabilis* HI4320 genome contains the *ids* genes (PMI2990–PMI2996) [15], [17], which we now propose to be an *hcp-vgrG* orphan effector operon. Three orphan *hcp-vgrG* operons, in addition to *idsA-F*, were also found: PMI0207–PMI0212, PMI1117–PMI1121, and PMI1332–PMI1324 (Fig. 6A and B). Examination of the predicted amino acid sequences reveals that the Hcp proteins of the *hcp-vgrG* effector operons are highly homologous, with the exception of the truncated protein encoded by PMI1332 (Fig. S6). The VgrG proteins have high percent amino acid sequence identity at the N-termini with decreasing homology beginning approximately 100 residues from the C-termini (Fig. S7). All of the *hcp-vgrG* effector operons have remarkably similar organization, where the first two genes of the effector operon are the *hcp*-*vgrG* pair followed by up to 7 genes that encode non-structural T6SS proteins with putative effector and immunity functions (Fig. 6B). Because the *ids* operon does not influence Dienes line formation in strain HI4320 but does in BB2000 [15], we examined the HI4320 genomic sequences upstream of all *hcp-vgrG* effector operons for promoter differences that could explain disparate expression. Alignment analyses of the five predicted promoter regions indicate a high degree of similarity (Fig. 6C).

Because the multiple sequence alignments show a high degree of conservation between the identified *hcp-vgrG* effector operon and the four orphan effector operons, we reasoned that all five *hcp* promoters could be functional. To test this hypothesis and to observe *hcp* expression during swarming, transcriptional *hcp* promoter-luciferase fusions were constructed for each *hcp-vgrG* effector operon and examined hourly for luminescence during swarming (Fig. 6D). A constitutive sigma-70 *em7* promoter (P_em7_) driving expression of *luxCDABE* [29] was included as a control. As the *P. mirabilis* strains containing the individual *hcp* promoter-luciferase fusions swarm outward across the swarm agar plate, the outer most edge of the bacterial swarm zone quantitatively emits the brightest light, in contrast to the constitutive *em7* construct that shows highest luminescence where cell density is the greatest (Fig. 6E). These findings indicate that actively swarming bacteria at the periphery are expressing the *hcp* promoter-luciferase fusion at higher levels than bacteria in the preceding swarm rafts (Fig. 6D and 6E). All five *hcp* genes and associated effector operons are expressed during multicellular swarming. We also found expression of all five reporter constructs occurs during liquid culture (Fig. S8). However, expression of the T6SS-related genes during batch culture is difficult to evaluate since the natural Dienes phenomenon and killing occurs exclusively in cells that have differentiated into swarm cells, neither of which occurs during culture in liquid medium. It is notable, that the constitutive control *em7* construct displays a linear relationship between luminescence and cell density as expected both in broth (Fig. S8) and on agar plates (Fig. 6E).

### The number and combination of *hcp-vgrG* effector operons does not dictate Dienes reaction

Because all of the HI4320 *hcp-vgrG* effector operons and the T6SS are expressed during swarming, we sought to determine a link between possession of *hcp-vgrG* effector operons and Dienes type using a collection of *P. mirabilis* clinical isolates. We hypothesized that variability in number and type of *hcp-vgrG* effector operons or the nucleotide variability within the same *hcp-vgrG* island available in a strain could dictate its ability to kill and overcome an opposing strain. To determine which *hcp-vgrG* effector operons are present, we performed multiplex PCR on chromosomal DNA of 16 *P. mirabilis* isolates, including HI4320 and BB2000. We screened for one effector gene localized to each of the *hcp-vgrG* effector operons and PMI0742 of the T6SS. PMI0742 and PMI0756 were present in all of the *P. mirabilis* isolates examined (Fig. S9), suggesting that the T6SS-linked PMI0750–PMI0758 effector operon may be the central *hcp-vgrG* effector operon for T6S-dependent killing. Due to these findings, we have termed the PMI0750–PMI0758 operon the primary *hcp-vgrG* effector operon (*pef*).

To determine if the presence of a specific *hcp-vgrG* effector operon has an effect on Dienes line formation, we examined the 16 *P. mirabilis* isolates against each other on swarm agar. While most isolates only merged with themselves and formed a Dienes line with all other isolates (red squares; Fig. S9, panel A), we found that some non-identical isolates do not form Dienes lines (green squares; Fig. S9, panel A). For example, *P. mirabilis* isolates DI120 and DE121 merge together and both are able to merge with KU140 but only DI120 will also merge with RZ130 and ST111, which are also able to merge with each other (Fig. S9). This clearly shows that the Dienes line can determine if two isolates are different. However, lack of a Dienes line does not indicate that two isolates are identical. Because these isolates have the same complement of *hcp-vgrG* effector operons (Fig. S9, panel C), the presence or absence of a specific *hcp-vgrG* effector operon does not determine Dienes type and thus dictate what strains will or will not form a Dienes line. We hypothesize that the Dienes phenomenon may involve other non-T6S factors in addition to the number or variability of *hcp-vgrG* effector operons a strain possesses. For example, previous studies have shown that contact-dependent growth inhibition (CDI) functions in *Escherichia coli* intercellular competition [30], [31], [32].

## Discussion

First described in 1946 [9], the macroscopic demarcation known as the Dienes line forms at the boundary between swarms of genetically related but non-identical isolates of *Proteus mirabilis* [10]. This phenomenon has been exploited as a powerful epidemiological tool in clinical microbiology laboratories to track outbreaks in hospital units [12], [13]. Here we show that Dienes line formation and the specificity of this direct cell-cell contact-dependent reaction precisely correlates with killing and this killing is dependent upon the single T6SS and its primary effectors. Upon initiation of swarming differentiation, the T6S apparatus is assembled and appears to fire when opposing swarms meet. Each strain is immune to killing itself. The dominant strain infiltrates deeply beyond the boundary of the two swarms and continues to assemble and discharge the T6SS. Dienes line formation, underlying killing, and immunity to killing, can be assigned to specific contiguous genes. These observations define the mechanism by which *P. mirabilis* uses contact-dependent delivery of effectors for interbacterial competition during a developmental process that coordinates multicellular behavior. Hence, we sought to use this multicellular model to study T6SS under conditions in which no genetic manipulation was necessary to activate assembly of the T6SS and trigger its firing. We propose that the *P. mirabilis* T6SS can serve as such a universal model to study this ubiquitous secretion system that mediates interbacterial competition.

That killing of an isogenic mutant is sufficient to produce a line of demarcation supports the notion that the biologically relevant non-isogenic bacterial T6SS interaction can result from strain A killing strain B, strain B killing strain A, or strain A and strain B killing each other. Our findings indicate that when two non-identical multicellular participants make contact, the failure to recognize the opposing cell as self is a result of the lethal action mediated by the effectors delivered by the T6SS. Thus, a lack of killing following cell-cell contact of two swarming populations active for T6SS-dependent killing equates to “recognition” of self. However, a great limitation of using mutant strains that are otherwise isogenic with the wild-type parent strain is that while the wild-type can kill a mutant strain, the complemented mutant will never kill the parent. But, by using non-isogenic, wild-type isolates, we confirmed that T6SS-mediated killing among multicellular bacteria is not unidirectional. That is, wild type swarm cells attempt to kill each other using T6SS-mediated injection of effectors immediately upon cell-cell contact. For example, when the swarms of strains HI4320 and BB2000 meet, a Dienes line forms. When a T6SS deletion mutant of HI4320 is substituted for its wild type strain, a Dienes line still forms. When a T6 deletion mutant of BB2000 is substituted for its wild type, a Dienes line again still forms. Only when HI4320ΔT6 is pitted against BB2000ΔT6, does no line form, indicating that HI4320 can kill BB2000 in a T6SS-dependent manner and BB2000 likewise can kill HI4320 in a T6SS-dependent manner. Only when the T6S systems of both strains are inactivated does the killing stop, preventing Dienes line formation. Thus, in the wild, while one strain may be dominant over another, all swarming strains of *P. mirabilis*, upon cell-cell contact, are likely preemptively deploying the T6SS to kill competitors, or for that matter, kill any neighboring cell within its own swarm. It is also possible that the benefit gained from preemptive deployment is to preserve a clonal population rather than to benefit by eliminating your competitor.

Interestingly, growth inhibition between colonies on agar medium has also been observed between different isolates of *P. aeruginosa*, *E. coli*, and *Salmonella spp.* [33], [34], [35]. However, this phenomenon, known as colony incompatibility, is thought to be due to bacteriophages, bacteriocins, or through production and excretion of antibiotics [33], [34]. A phenomenon similar to colony inhibition has been noted in *E. coli* [30], [32], suggesting universality of multicellularity, cell-cell contact, and competition with non-identical strains of the same species [8]. However, until now, an appropriate multicellular system has not been described that would aid in answering the open questions of why bacteria use contact-dependent delivery of effectors to eliminate one's neighbors [8]. Would contact dependence allow discrimination or are T6SS-dependent interactions involved in developmental processes such as multicellularity or organized communities [8]?

Recent work [15], described the *ids*, or “identify of self” genes, responsible for self-recognition in *P. mirabilis* BB2000 and proposed that the function of the *ids* operon could be related to a diffusible bar code scanning mechanism of recognition that precedes the decision to attack [16]. In that work, T6S was ruled out because, while *idsA* and *idsB* were noted to have some homology to *hcp* and *vgrG*, the authors were unable to identify any genes near the *ids* locus that encode the structural components of the T6SS [15], [16]. In our study, we identified not only the T6SS genes themselves but also, immediately adjacent, specific genes in the primary *hcp-vgrG* effector operon as required to kill the immunity-deficient mutant 9C1. Because T6S-dependent killing occurs when two non-identical strains make contact, we hypothesized that the genes downstream of *hcp* and *vgrG* encode effectors that are injected by the T6SS into the recipient bacterium's periplasm that ultimately leads to death for the target cell. By interrogating the *P. mirabilis* genome, we found not only the *pef* operon linked to the T6SS, but four additional conserved orphan *hcp-vgrG* effector operons (including the *ids* operon), which are unlinked to the single T6SS in *P. mirabilis*. Our finding that *ids* is an orphan T6SS effector operon, found only in a minority of strains, explains why the genes encoding structural components of the T6SS apparatus were not identified nearby the *ids* genes [16]. In addition, a limitation of the bar code scanner or self-recognition model [16] proposed for the Dienes phenomenon is that the mere presence of a line between swarm populations is not sufficient to identify the dominant or susceptible strain.

While it is unclear that the specific number or combination of effector operons controls whether or not a multicellular population of *P. mirabilis* forms a Dienes line with another swarm, it is clear that the Dienes reaction requires the T6SS to be active in only one of the two populations. Therefore, we speculate that certain strains may possess immunity to specific Hcp-VgrG effector operons while lacking the ability to kill with the respective operon. While we have shown that HI4320 and BB2000 are capable of killing one another and, thus, HI4320 is not immune to BB2000, the existence of multiple *idsE* homologs has been proposed to provide HI4320 immunity against BB2000 [15]. Yet, it was not reported whether BB2000 or other strains that are not fully sequenced and annotated, like HI4320, also possess multiple *idsE* copies. The existence of multiple “orphan” *hcp-vgrG* effector operons within a bacterial genome is not uncommon [36]. One study [1] discovered three IcmF-associated (TIGR0334) homologous protein (IAHP) loci in the *Pseudomonas aeruginosa* PAO1 genome that encode *hcp* and *vgrG* homologs, one of which has been proposed to be involved in virulence [1]. The organization of orphan T6SS effector operons with cognate *hcp* and *vgrG* genes that are not encoded nearby the T6SS genes has been observed in a number of bacterial genomes for which sequences are available [25], [36]; our findings of four orphan T6SS effector operons in a single genome suggests that novel lethal effector and immunity genes may be readily identified in other bacteria, linked with their respective *hcp*-*vgrG* pair. Further, our findings demonstrate that, despite high (>90%) amino acid sequence identity between the multiple Hcp and VgrG proteins in HI4320, the orphan pairs are unable to substitute for the primary Hcp and VgrG to provide the ability to kill the immune-deficient 9C1 strain.

Immunity to self-killing (*i.e.*, “recognition”) strongly supports the preemptive model for T6SS-dependent antagonism and also explains why isogenic wild-type strains do not normally kill one another when in cell-cell contact during multicellular cooperation of specialized swarm cells. Our findings clearly show that it is loss of immunity that allows an otherwise isogenic mutant strain to be killed, however, by definition, isogenic wild type strains do not kill themselves because they would only exist in the absence of the multicellular behavior that has evolved to use the T6SS to gain a specific advantage. Indeed, it is difficult to explain how loss of immunity in one isogenic pair is sufficient to observe naturally expressed T6SS lethality when two strains are mixed and co-inoculated unless the secretion system is preemptively armed to fire upon direct cell-cell contact.

Our data support the idea that one gene from the primary *hcp-vgrG* effector operon, *pefE* (PMI0756), encodes a dual function protein that is necessary to confer immunity to preemptive attack by cognate T6SS-delivered effectors. This protein is also required for the lethal activity of the respective primary operon-encoded effectors. Indeed, we have shown that PefE alone can confer immunity to killing by the primary Hcp-VgrG delivered effectors, while *pefE* and the entire *pef* operon are required for T6SS-mediated lethality. Future studies will reveal if each of the T6SS-dependent *pef* gene products delivered by the T6SS function independently as cognate pairs [37] or perhaps assemble in the target cell to form a multi-protein complex or functional effector “package”. We speculate the dual function of PefE is a failsafe mechanism that has evolved to prevent self-killing in the event that loss of immunity occurs. It is notable that insertions within the identified *hcp-vgrG pef* effector operon, in any gene upstream or downstream of PMI0756, abrogates the ability to kill and form a Dienes line with 9C1 but does not cause loss of immunity to wild-type, which suggests that there is a second promoter driving expression of the failsafe *pefE*.

We cannot rule out the possibility that *pefE* encodes a protein that provides an immune function by binding a single cognate effector. It is possible that the *pef* ‘effectors’ work sequentially, and PefE inactivates a single toxic protein required for antagonistic effects. It could also be possible that a complex representing multiple *pef* gene products assembles in the target cell, and PefE interferes with the formation of the complex. Alternatively, the mechanism for immunity could also be to block or prevent the preemptive T6SS strike. The dual-function of PefE (that is, immunity and killing) suggests it may also act as a chaperone or otherwise interact with another protein to provide killing function for the attacker cell.

Interestingly, we have been unable to identify toxin-immunity pairs within the HI4320 genome orthologous to those effectors currently described in *P. aeruginosa* [6]. It is possible that the currently described effector-immunity pairs from *P. aeruginosa* may be accessory effectors as the Pseudomonads have large genomes to support a generalist lifestyle, unlike *Proteus* with a more compact genome. However, our findings suggest that T6S-mediated interbacterial competition is fundamentally similar between these microorganisms. In support of this, we have identified a PefE orthologous protein encoded within the HSI-1 T6SS locus of *P. aeruginosa*. Since PefE appears to provide an immune function in *Proteus*, and the ortholog is encoded within a T6SS locus, we reason it may perform a similar function when *P. aeruginosa* naturally expresses its T6SS for antagonizing a natural competitor. Furthermore, the fundamental similarity between T6SS's could also be represented by lysozyme-like effectors; PMI0752 (*pefA*) encodes a putative bacteriophage-like gp5 lysozyme domain. Bacteriophage, including T4 that is structurally related to the T6SS, requires lysozyme activity to breach the cell wall of the target bacterial cell and to inject their DNA, yet it remains unresolved how the T6SS puncturing device would deliver lethal effectors without lysozyme activity. Since the T6SS appears to target bacterial cells, we hypothesize that the T6SS, like bacteriophage puncturing devices, also requires lysozyme activity to deliver a lethal payload. Consistent with the T6SS requiring lysozyme activity, both *P. aeruginosa* and *V. cholerae* have been shown to encode T6SS effectors with lysozyme activity [6], [23].

Our model has provided evidence to answer a number of open questions regarding the role for bacterial T6SS during competition, in general, and its potential relationship to multicellularity [8]. Studying T6S in a multicellular model has overcome a number of limitations that arise from studying the T6SS in bacteria under conditions where the bacteria being studied do not normally express the T6SS, do not require cell-cell contact, or the natural competitor is not known. For example, it has been shown that in *V. cholerae*, a planktonic bacterium, the global regulator RpoN positively regulates T6SS-dependent *hcp* and *vgrG* expression, but has no effect on expression of the core, cognate T6SS structural genes, including *vipA* [38]. Therefore, forced expression of T6SS activity may incompletely activate T6S-dependent processes and make interpretations of results difficult. Furthermore, if the bacterial physiology dictates repression of T6SS activity, then it would be difficult to distinguish true immunity from the absence of an effector target that might occur due to incomplete or forced expression of T6SS activity during times when the bacteria do not normally express that function. Forced expression of T6SS activity could also lead to recognition [15], [16] and decision-based models [20] for T6S function due to incomplete activation in one or more bacterial strains.

Several lines of evidence in the present study point toward a preemptive model for T6SS-mediated antagonism that is intimately linked to a cellular differentiation pathway in bacteria that culminates in the formation of specialized cells that function to coordinate a cooperative and multicellular behavior. The preemptive model for T6S in bacteria is also consistent with the generally accepted notion that T6SS-dependent activity is cell-cell contact-dependent and deployment of the pre-assembled contractile puncturing device into the target cell is extremely rapid [22]. Because T6S is dependent on cell-cell contact, it would seem beneficial for bacteria exhibiting contact-dependent multicellular behavior to employ the T6SS to discriminate, “recognize”, and kill competitors rather than indiscriminately secrete bactericidal agents when competing for resources in their natural habitats. Indeed, our findings indicate that function of theT6SS and the *pef* gene products encoded by the primary *hcp-vgrG* effector operon are coordinately linked to multicellular swarming in *P. mirabilis*. It is uncertain whether the interdependence of T6SS-dependent killing and multicellularity truly functions for bacterial competition or simply to maintain homogenous population during the development of multicellularity. We hypothesize that during multicellular swarming of *P. mirabilis*, the T6SS is continuously functioning in a preemptive manner in actively swarming populations to prevent cheaters from benefiting from the cooperative behavior [39], [40]. The T6SS may restrict non-identical populations from swarming within each other's multicellular population. Thus, kin selection may be advantageous to preserve uniform bacterial swarming populations to maximize cooperation during migration to new resources or away from predators in their natural habitat.

## Materials and Methods

### Bacterial strains, transposon screen, and identification of mutants

*P. mirabilis* HI4320 was cultured from the urine of a nursing home resident with catheter-associated bacteriuria [41]. A transposon library of 1920 *P. mirabilis* HI4320 insertion mutants [42] was screened for Dienes line formation on swarm agar [lysogeny broth (LB) medium containing NaCl (10 g/L) and 1.5% agar] by spotting the plate with wild-type HI4320 and mutants (for example see Fig. 1C). For the transposon screens, bacteria were plated by spotting 5 µl of an overnight LB broth culture onto swarm agar, incubated at 37°C, and observed within 18 h. To identify genes containing insertion mutations, arbitrary PCR on genomic DNA was used to amplify the 3′ end of the transposon and flanking chromosomal DNA as previously described [42]. PCR products were cloned into the pCR2.1-TOPO vector (Invitrogen), maintained in *E. coli* TOP10 (Invitrogen), and sequenced to identify the transposon insertion site. *P. mirabilis* HI4230 mutants 0754*::kan*, 0755*::kan*, and 0758*::kan* were constructed using the TargeTron kit (Sigma-Aldrich), and T6SS mutants were constructed by allelic replacement. Antibiotics were added as necessary at the following concentrations: kanamycin, 25 µg/ml; ampicillin, 100 µg/ml; and chloramphenicol, 20 µg/ml; for pBAD constructs, 10 mM L-arabinose was added to swarm plates to induce pBAD VipA::sfGFP.

### Complementation

Dienes line formation between *P. mirabilis* mutant 9C1 and the identified transposon insertion mutants was restored by complementing the insertion mutants with pGEN containing a DNA fragment carrying the T6SS-linked *hcp-vgrG* effector operon (PMI0750–PMI0758). This 9-kb fragment was PCR amplified with Phusion High-Fidelity Polymerase (Thermo-Fisher Scientific), digested with restriction enzymes SphI and NotI (New England Biolabs), and ligated to the linearized pGEN-MCS vector [43]. The resulting construct was transformed into the *P. mirabilis* insertion mutants. Individual genes and partial operons were amplified from HI4320 genomic DNA and cloned under control of the arabinose inducible promoter in pBAD-MycHisA (Invitrogen).

### Bacterial killing assays

A suspension (5 µl) containing a 1∶1 ratio of strains was spotted onto swarming agar. Following incubation overnight at 37°C, the entire swarm was collected from the agar plate, serially diluted, and plated on LB medium containing NaCl (0.5 g/L) and 1.5% agar containing Amp (100 µg/ml) for the parental strain or kanamycin (25 µg/ml) for the 9C1 mutant to determine CFU/ml. The output ratio was compared to the input ratio to quantify killing of mutant 9C1. The *cat* gene was cloned into linearized pGEN-MCS vector and transformed into the transposon mutants to distinguish these (Amp^R^Cam^R^) from mutant 9C1 containing pGEN-MCS (Amp^R^). 9C1 killing is reported as [(CFU of test strain/CFU of 9C1)_output_]/[(CFU of test strain/CFU of 9C1)_input_].

### Live-cell microscopy

To capture live images of Dienes line formation, *P. mirabilis* HI4230 and the 9C1 mutant were spotted (5 µl) from overnight LB cultures onto opposite sides of an agar plate, allowed to swarm at 37°C, and imaged directly on swarming agar under phase contrast and fluorescence microscopy. For fluorescence studies, *P. mirabilis* HI4230 was transformed with a plasmid expressing sfGFP. PMI0749, which encodes *vipA*, was amplified from HI4230 chromosomal DNA; sfGFP was amplified by PCR and VipA was fused with sfGFP, separated by a DNA linker encoding 3×Ala 3×Gly, and cloned into plasmid pBAD-myc-HisA [22]. pGEN encoding Red fluorescent protein (dsRED) [44] was transformed into mutant 9C1. For vital staining, 5 µl of 5 µM SYTO 9 (Invitrogen) was applied directly to bacteria on swarm agar for 5 min prior to visualization. Microscopy experiments were performed in the Center for Live Cell Imaging (CLCI) at the University of Michigan Medical School using an Olympus IX70 inverted microscope with FITC and Texas Red filter sets (Olympus). Images were collected using a CoolSNAP HQ2 14-bit CCD camera (Photometrics). All devices were controlled through Metamorph Premier v6.3 software (Molecular Devices). Initial analysis of the imaging data and the preparation of image overlays, montages and movies, were performed using Metamorph v7.7 software. If necessary, deconvolution of the images was performed using Huygens v4.1 software (Scientific Volume Imaging BV).

### Luciferase-gene expression

To observe expression of the multiple *hcp-vgrG* effector operons during swarming, a 500-bp fragment immediately upstream of the translational start of *hcp* was PCR amplified from chromosomal HI4320 DNA with EasyA Polymerase (Stratagene), digested with restriction enzymes PmeI and SnaBI (New England Biolabs), and ligated into pGEN-*lux* [29] to create transcriptional *hcp* promoter-luciferase fusions. The constitutive reporter P_em7_ *-lux* and a promoterless construct (P_neg_-*lux*) were used as controls [29]. LB cultures were incubated overnight and diluted 1∶100 into fresh LB medium containing ampicillin (100 µg/ml). OD_600_ of a 200 µl sample volume was read every h as well as the luminescent emission (100 µl sample volume) using a Synergy HT plate-reader operating KC4 software (Bio-Tek, Winooski, VT). Luminescence was plotted as a function of cell density as measured by OD_600_ over time. For swarming studies with the *hcp* promoter-luciferase fusions, 5 µl of an overnight bacterial LB culture was spotted in the center of a swarm agar plate. Following incubation at 37°C, luminescent emission was captured using the ChemiDoc XRS system (Bio-Rad). Further analysis was conducted to obtain density plots of the luminescence using the Discovery Series Quantity One software (Bio-Rad).

### Sequence alignment

Nucleotide sequences of *P. mirabilis* HI4320 genes were obtained from the Kyoto Encyclopedia of Genes and Genomes (KEGG) [45] and saved as SeqBuilder files (DNASTAR). Sequence files were entered into MegAlign (DNASTAR) and subjected to multiple alignment using Clustal W [46]. Alignment data of the predicted promoters of the five *hcp-vgrG* effector operons and PMI0742 are presented as a phylogenetic tree to view predicted evolutionary relationships.

### Dienes typing

Sixteen *P. mirabilis* clinical isolates [47] were examined and recorded for Dienes line formation with one another by spotting a 5 µl volume from a bacterial overnight culture onto swarm agar and incubating at 37°C for 18 h. *P. mirabilis* HI4320 mutant 9C1, transposon mutant 14H2, which has a disruption in PMI0742 of the T6SS, and mutants of *idsB* and *idsD*, PMI2991 and PMI2993, respectively, were also included in these tests as controls.

### Multiplex PCR

Multiplex PCR (Qiagen) was performed by amplifying the chromosomal DNA of 16 *P. mirabilis* isolates for the presence of an effector gene representative of each of the *hcp-vgrG* effector operons. Primers were designed for *P. mirabilis* HI4230 genes PMI0210, PMI1120, PMI0756, PMI1329, and PMI2993 of each *hcp-vgrG* effector operon which resulted in variable sized PCR products; 250-bp, 350-bp, 600-bp, 850-bp, and 1-kb, respectively. PMI0742 of the T6SS was also included and amplified with forward (F) primer, 5′CTCAAGAGCCGGTGATCCATCCTGAAAAAC3′ and reverse (R) primer 5′GTAATTGTCTTGGTGCAGCCGAAAGTG3′ resulting in a 450-bp PCR product. PMI0210 was amplified with F primer 5′TTATTGCTTGGCGAGGCTCTCAGG3′ and R primer 5′GCCAAAGCTAAAGCTCCTCCTAAGCTATG3′, PMI0756; F primer 5′TGCTTAAAACCGAAAGAACAAGGGATGC3′ and R primer 5′CCATTCCAACACTGTAAACGGTAGTC3′, PMI2993; F primer 5′AATTAACGGAACAAATAGTACCAAATCTGC3′ and R primer 5′GCCAAGCCGCTGTGATAACCAAC3′, PMI1120; F primer 5′GCGTCAGCAGGTCTATGAATATAG3′ and R primer 5′CATAACGATAACGGGTGGTTTTTC3′, PMI1329; F primer 5′TATTGTTGTTTGGCGAGGAACGGC3′ and R primer 5′TGAGTGGTCTCCACCACCAGTTAC3′

## Supporting Information

Figure S1 **Live cell microscopy shows cell death occurs at the Dienes line.** (**A**) Brightfield microscopy of the macroscopic Dienes boundary on a swarm agar plate 4 h after contact between opposing swarms of *P. mirabilis*. Viability was assessed directly on the agar plate using fluorescence microscopy by placing a 5 µl droplet of SYTO 9 fluorescent green nucleic acid stain on (**B**) the center of the Dienes line, and on (**C–F**) bacteria located on either side of the boundary. Brightfield and fluorescence microscopy showing SYTO 9 staining of the sensitive swarm side (**C**) and (**D**) that lacks immunity against the T6SS effectors delivered by the infiltating swarm cells originating from the dominant, wild-type HI4320 swarm side (**E**) and (**F**). In (A) the dashed line (white) represents the center of the Dienes line and the merge point between the opposing swarms (black arrows). Unoccupied areas of the agar surface are indicated with an asterisk.
(JPG)

Figure S2 **Localization of sfGFP expressed as a translational fusion to the T6SS sheath from** ***P. mirabilis*** **HI4320.** Epifluorescence visualized from live, actively swarming *P. mirabilis* expressing (**A–C**) VipA::sfGFP or (**D–F**) sfGFP under control of the pBAD arabinose-inducible promoter. For these experiments wild-type *P. mirabilis* HI4320 (green) (A,D) infiltrating susceptible 9C1 expressing dsRED (red) (B,E) were visualized directly on an agar plate containing 10 mM L-arabinose to induce expression of sfGFP constructs. Merged images (C,F). Fusion of sfGFP to the T6SS sheath encoded by PMI0749 (VipA) causes punctuate localization in (A) fluorescence emitted by sfGFP as compared to the diffuse fluorescence observed in (D) when sfGFP is independently expressed.
(TIF)

Figure S3 **Fusion of the putative lysozyme effector with the signal peptide from** ***P. mirabilis*** **HI4320 PhoA and expression of PhoA::0752 in** ***P. mirabilis*** **HI4230 and the 9C1 mutant does not affect viability during swarming.** (**A**) *E. coli* containing pBAD, pBAD0752, pBAD PhoA::0752 were diluted and plated on LB agar containing high salt (0.17 M NaCl) with (+) and without (−) 10 mM L-arabinose. Decreased viability was observed only when PMI0752 is both arabinose-induced and expressed with the PhoA signal peptide. The constructs expressed in (**B**) wild-type HI4320 and (**C**) the sensitive 9C1 mutant on low salt (0.008 M NaCl) or high salt (0.17 M NaCl) LB agar with (+) and without (−) 10 mM L-arabinose. No difference is observed between the wild-type HI4320 parent strain and the 9C1 immune-defective mutant.
(TIF)

Figure S4 **Disruption of PMI0754 or PMI0755 from the primary** ***hcp*** **-** ***vgrG*** **effector operon in** ***P. mirabilis*** **HI4320 abolishes Dienes line formation with 9C1.** *P. mirabilis* mutants in genes PMI0754 (*0754::kan*) and PMI0755 (*0755::kan*) containing pBAD empty vector (pBAD), pBAD0754 or pBAD0755, or pBAD0754–0758 inoculated opposing 9C1 onto swarm agar (−) or agar plates containing 10 mM L-arabinose (+). HI4320 containing pBAD was included as a control. *0754::kan* and *0755::kan* are unable to form a line with 9C1 unless complemented by the disrupted gene plus the remainder of the primary effector operon (black arrows). Note the presence of a partial Dienes line between HI4320 and *0755::kan* and between *0755::kan* pBAD and induced *0755::kan* pBAD0754–58 (white arrows).
(TIF)

Figure S5 **Disruption of PMI0742 abolishes T6SS function.** *P. mirabilis* HI4320 mutant 14H2 has a transposon insertion in the T6SS gene PMI0742 and cannot kill susceptible mutant 9C1. Targeted disruption of PMI0742 (0742*::kan*) also abolishes the ability of HI4320 to kill and form a Dienes line with mutant 9C1.
(TIF)

Figure S6 **The Hcp amino acid sequences of the** ***hcp-vgrG*** **effector operons in** ***P. mirabilis*** **HI4320 are highly conserved.** Alignment of Hcp amino acid sequences encoded by PMI0207, PMI0750, PMI1117, PMI2990, and PMI1332. The Hcp protein sequences of four of the *hcp-vgrG* effector operons are highly conserved; shaded in green. Boxed amino acids shaded white indicate divergences. PMI1332 is truncated in comparison to the other Hcp proteins by approximately 81 amino acids and as a result has decreased homology to the other Hcp proteins.
(TIF)

Figure S7 **The VgrG amino acid sequences of the** ***hcp-vgrG*** **effector operons in** ***P. mirabilis*** **HI4320 are conserved at the N-terminus and highly variable at the C-terminus.** The N-termini of the VgrG homologs encoded by the 5 *hcp-vgrG* effector operons in HI4320 are highly conserved beginning at the N-termini with decreasing homology beginning approximately 100 residues before the C-termini. Bar colors represent conservation at each position, red; conservation among all 5 VgrG proteins, orange; 4 VgrG proteins share the same amino acid, green; conservation between 3 VgrG proteins, light blue; shared by 2 VgrG and, dark blue; no conservation at that position.
(TIF)

Figure S8 ***In vitro*** **expression of the** ***hcp-vgrG*** **effector operons in** ***P. mirabilis*** **HI4320 in liquid suspension.** *P. mirabilis* strain HI4320 expressing *hcp* promoter-luciferase transcriptional fusions were observed over time during growth in LB medium. Luminescence is plotted as a function of cell density (OD_600_) as measured over time.
(TIF)

Figure S9 **The presence or absence of specific** ***hcp-vgrG*** **effector operons within 16** ***P. mirabilis*** **clinical isolates does not designate Dienes type.** (**A**) A total of 16 *P. mirabilis* clinical isolates (BR2528, DI120, DE121, HA139, KU140, MA126, MC118, PL105, RO106, RZ130, ST111, TA507, HU1069, BA6163, BB2000, and HI4320) were examined against each other on swarm agar for formation of a Dienes line. *P. mirabilis* HI4230 mutant 9C1, T6SS mutant 14H2, and *ids* mutants 2991 (*idsB*) and 2993 (*idsD*) were also tested against the 16 *P. mirabilis* isolates and each other for a line of demarcation. Red boxes indicate Dienes line formation, green boxes indicate the swarms merged without visible demarcation, and “w” indicates formation of a weak Dienes line. Strains never form a Dienes line with themselves. (**B**) DNA gel electrophoresis following Multiplex PCR on chromosomal DNA of the 16 *P. mirabilis* isolates. Primers were designed to amplify one conserved unique gene from each of the 5 *hcp-vgrG* effector operons and PMI0742 of the T6SS based on the *P. mirabilis* HI4230 genome. PCR product sizes for PMI0210, PMI1120, PMI0742, PMI0756, PMI1329, PMI2993 are approximately 250-bp, 350-bp, 450-bp, 600-bp, 850-bp, and 1-kb, respectively. (**C**) Chart showing presence of *hcp-vgrG* among the *P. mirabilis* isolates: BR; BR2528, DI; DI120, DE; DE121, HA; HA139, KU; KU140, MA; MA126, MC; MC118, PL; PL105, RO; RO106, RZ; RZ130, ST; ST111, TA; TA507, HU; HU1069, BA; BA6163, and BB; BB2000, HI; HI4320. Green boxes indicate presence of gene (PMI0210, PMI0742, PMI0756, PMI1120, PMI1329, PMI2993) within strain listed and red boxes indicate the gene was undetected within the strain listed. Δ indicates a mutation of the gene in mutant strains; *P. mirabilis* HI4230 mutant 9C1, T6SS mutant 14H2, and *ids* mutants PMI2991 and PMI2993.
(TIF)

Movie S1 **Merger of opposing swarming populations of** ***P. mirabilis*** **.** Live-cell fluorescence microscopy captures the merging of multicellular swarming populations of *P. mirabilis* wild-type HI4230 and mutant 9C1. *P. mirabilis* HI4230 constitutively expressing green fluorescent protein (GFP) and cultures of mutant 9C1 constitutively expressing dsRed were spotted on opposite sides of a swarm agar plate and were observed in real-time under brightfield and fluorescence microscopy as the bacterial strains swarmed toward one another. The strains clearly merge and seamlessly interdigitate as cells from opposing swarms first come into direct cell-cell contact immediately upon merging. Single channel fluorescence, brightfield, and merged images are presented.
(MOV)

Movie S2 **Live-cell fluorescence microscopy capturing the infiltration of** ***P. mirabilis*** **HI4320 within the opposing 9C1 mutant swarm following merging of the two populations.** Strain HI4320 expressing VipA::sfGFP under control of pBAD is observed infiltrating deeply into the opposing 9C1 swarm labeled with dsRED. Images are taken in real-time approximately 3 mm beyond the merge point within the 9C1 swarm. Only individual wild-type HI4320 swarmer cells are observed in the 9C1 swarm and appear to maintain the ability to rapidly migrate within the opposing swarm. Swarms were visualized on swarm agar containing 10 mM L-arabinose to induce expression of the VipA::sfGFP translational fusion. Single channel fluorescence, brightfield, and merged images are presented.
(MOV)

Movie S3 **Live-cell microscopy of the merge between two identical** ***P. mirabilis*** **HI4320 swarms.** Strain HI4320 expressing VipA::sfGFP under control of pBAD is observed merging with HI4320 expressing dsRED. The images were taken at the boundary between the red and green swarms as the two swarms merge. Equal penetration into the opposing swarm is observed between identical swarms expressing different colored fluors. Swarms were visualized on swarm agar containing 10 mM L-arabinose to induce expression of the VipA::sfGFP translational fusion. Single channel fluorescence, brightfield, and merged images are presented. Also see Movie S4.
(MP4)

Movie S4 **Live-cell microscopy of the merge between dominant** ***P. mirabilis*** **HI4320 and susceptible 9C1 swarms.** Strain HI4320 expressing VipA::sfGFP under control of pBAD is observed merging with mutant 9C1 expressing dsRED. The images were taken at the boundary between the red and green swarms as the two swarms merge. The susceptible 9C1 mutant swarm (red) is unable to penetrate the dominant parental wild-type HI4320 swarm (green) due to an immune-deficiency. Swarms were visualized on swarm agar containing 10 mM L-arabinose to induce expression of the VipA::sfGFP translational fusion. Single channel fluorescence, brightfield, and merged images are presented.
(MP4)

Movie S5 **Visualization of** ***P. mirabilis*** **T6SS-activity within an opposing 9C1 mutant swarm.** Strain HI4320 expressing VipA::sfGFP under control of pBAD is observed during infiltration deep within the 9C1 swarm. The appearance and disappearance of distinct puncta of green fluorescence indicates active and rapid assembly and firing of the T6SS during attack on the susceptible 9C1 cells. Swarms were visualized on swarm agar containing 10 mM L-arabinose to induce expression of the VipA::sfGFP translational fusion. The FITC (green) fluorescence is presented alone to maximize visualization of T6SS activity.
(MOV)

Table S1 **The** ***P. mirabilis*** **T6SS is conserved with those in** ***V. cholerae*** **and** ***P. aeruginosa*** **.**
(XLSX)
