# Supplementary figures and images for: Multicellular Bacteria Deploy the Type VI Secretion System to Preemptively Strike Neighboring Cells
Source: PLoS Pathog. 2013 Sep 5;9(9):e1003608. doi: 10.1371/journal.ppat.1003608 (PMC3764213; doi:10.1371/journal.ppat.1003608)

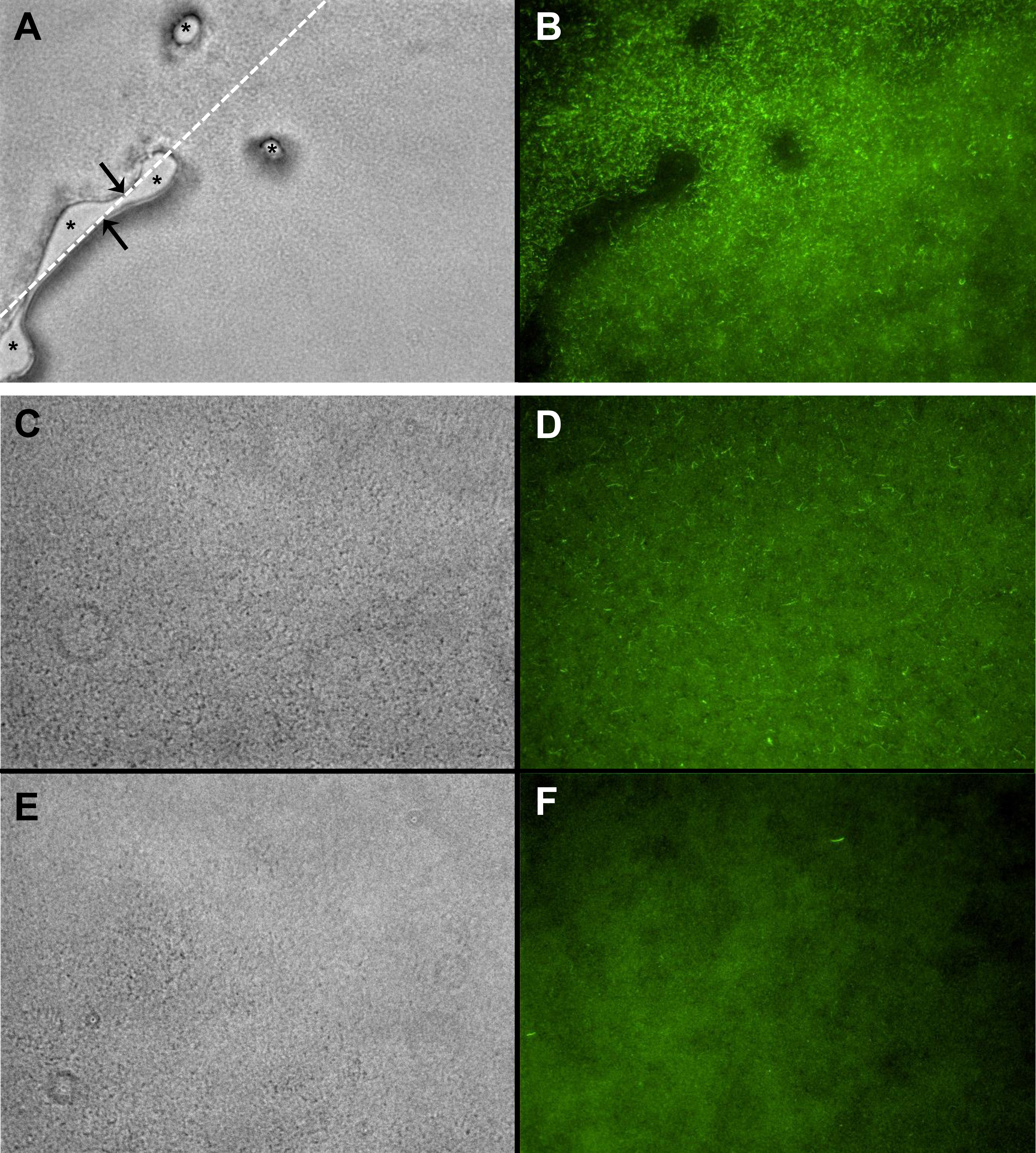

Supplement: Figure S1 — Live cell microscopy shows cell death occurs at the Dienes line. (A) Brightfield microscopy of the macroscopic Dienes boundary on a swarm agar plate 4 h after contact between opposing swarms of P. mirabilis. Viability was assessed directly on the agar plate using fluorescence microscopy by placing a 5 µl droplet of SYTO 9 fluorescent green nucleic acid stain on (B) the center of the Dienes line, and on (C–F) bacteria located on either side of the boundary. Brightfield and fluorescence microscopy showing SYTO 9 staining of the sensitive swarm side (C) and (D) that lacks immunity against the T6SS effectors delivered by the infiltating swarm cells originating from the dominant, wild-type HI4320 swarm side (E) and (F). In (A) the dashed line (white) represents the center of the Dienes line and the merge point between the opposing swarms (black arrows). Unoccupied areas of the agar surface are indicated with an asterisk. (JPG) [file ppat.1003608.s001.jpg]

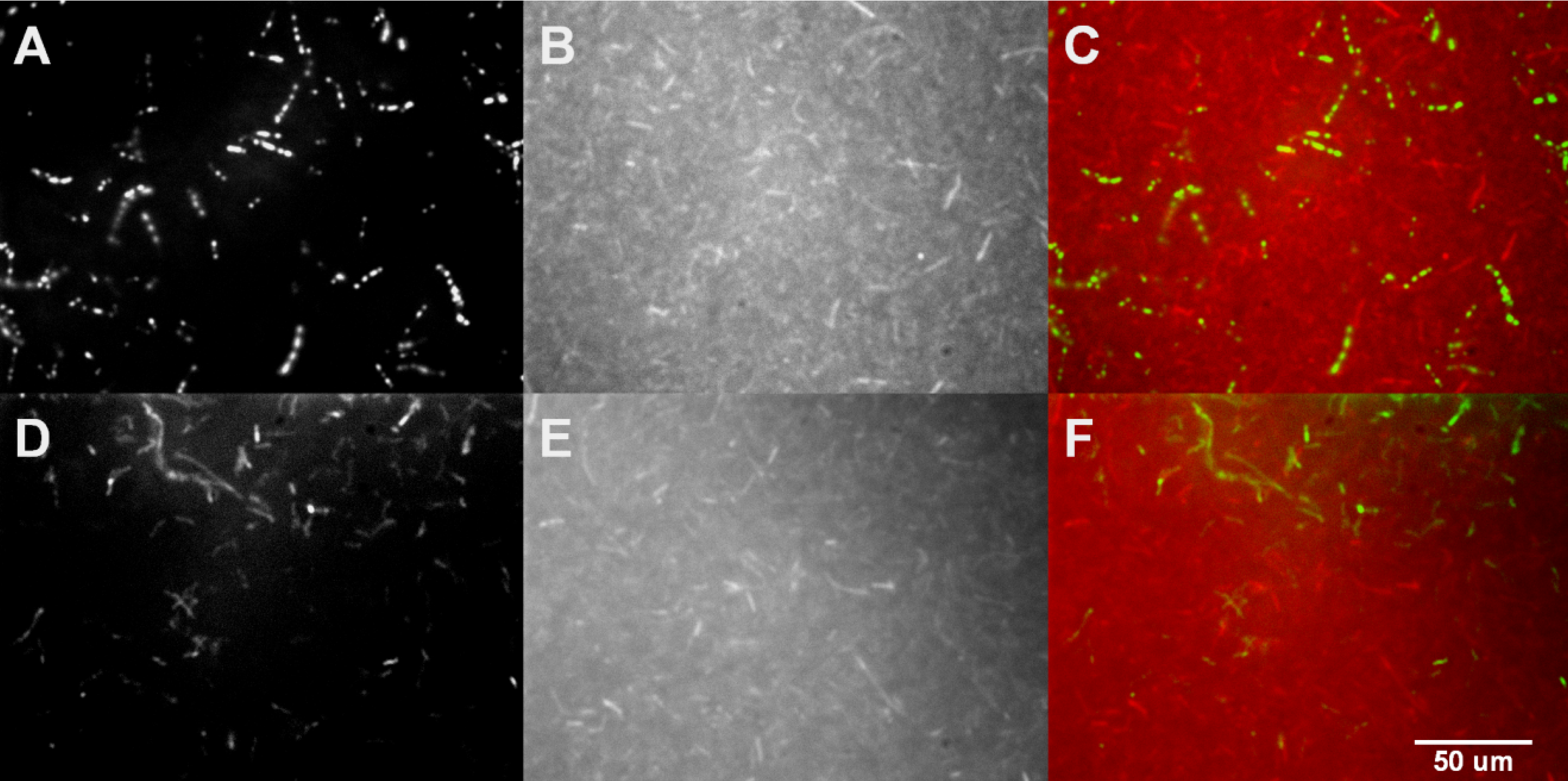

Supplement: Figure S2 — Localization of sfGFP expressed as a translational fusion to the T6SS sheath from P. mirabilis HI4320. Epifluorescence visualized from live, actively swarming P. mirabilis expressing (A–C) VipA::sfGFP or (D–F) sfGFP under control of the pBAD arabinose-inducible promoter. For these experiments wild-type P. mirabilis HI4320 (green) (A,D) infiltrating susceptible 9C1 expressing dsRED (red) (B,E) were visualized directly on an agar plate containing 10 mM L-arabinose to induce expression of sfGFP constructs. Merged images (C,F). Fusion of sfGFP to the T6SS sheath encoded by PMI0749 (VipA) causes punctuate localization in (A) fluorescence emitted by sfGFP as compared to the diffuse fluorescence observed in (D) when sfGFP is independently expressed. (TIF) [file ppat.1003608.s002.tif]

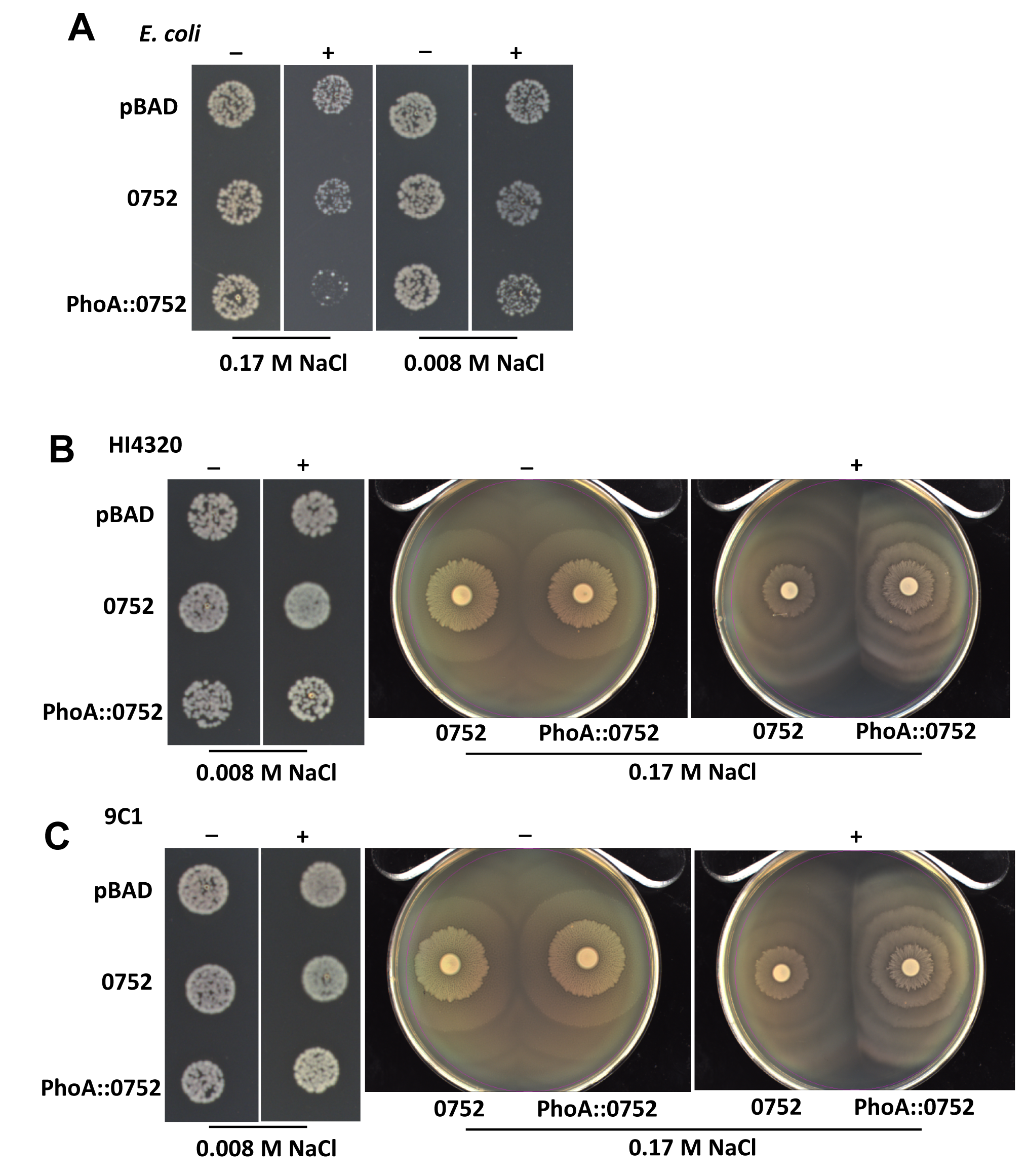

Supplement: Figure S3 — Fusion of the putative lysozyme effector with the signal peptide from P. mirabilis HI4320 PhoA and expression of PhoA::0752 in P. mirabilis HI4230 and the 9C1 mutant does not affect viability during swarming. (A) E. coli containing pBAD, pBAD0752, pBAD PhoA::0752 were diluted and plated on LB agar containing high salt (0.17 M NaCl) with (+) and without (−) 10 mM L-arabinose. Decreased viability was observed only when PMI0752 is both arabinose-induced and expressed with the PhoA signal peptide. The constructs expressed in (B) wild-type HI4320 and (C) the sensitive 9C1 mutant on low salt (0.008 M NaCl) or high salt (0.17 M NaCl) LB agar with (+) and without (−) 10 mM L-arabinose. No difference is observed between the wild-type HI4320 parent strain and the 9C1 immune-defective mutant. (TIF) [file ppat.1003608.s003.tif]

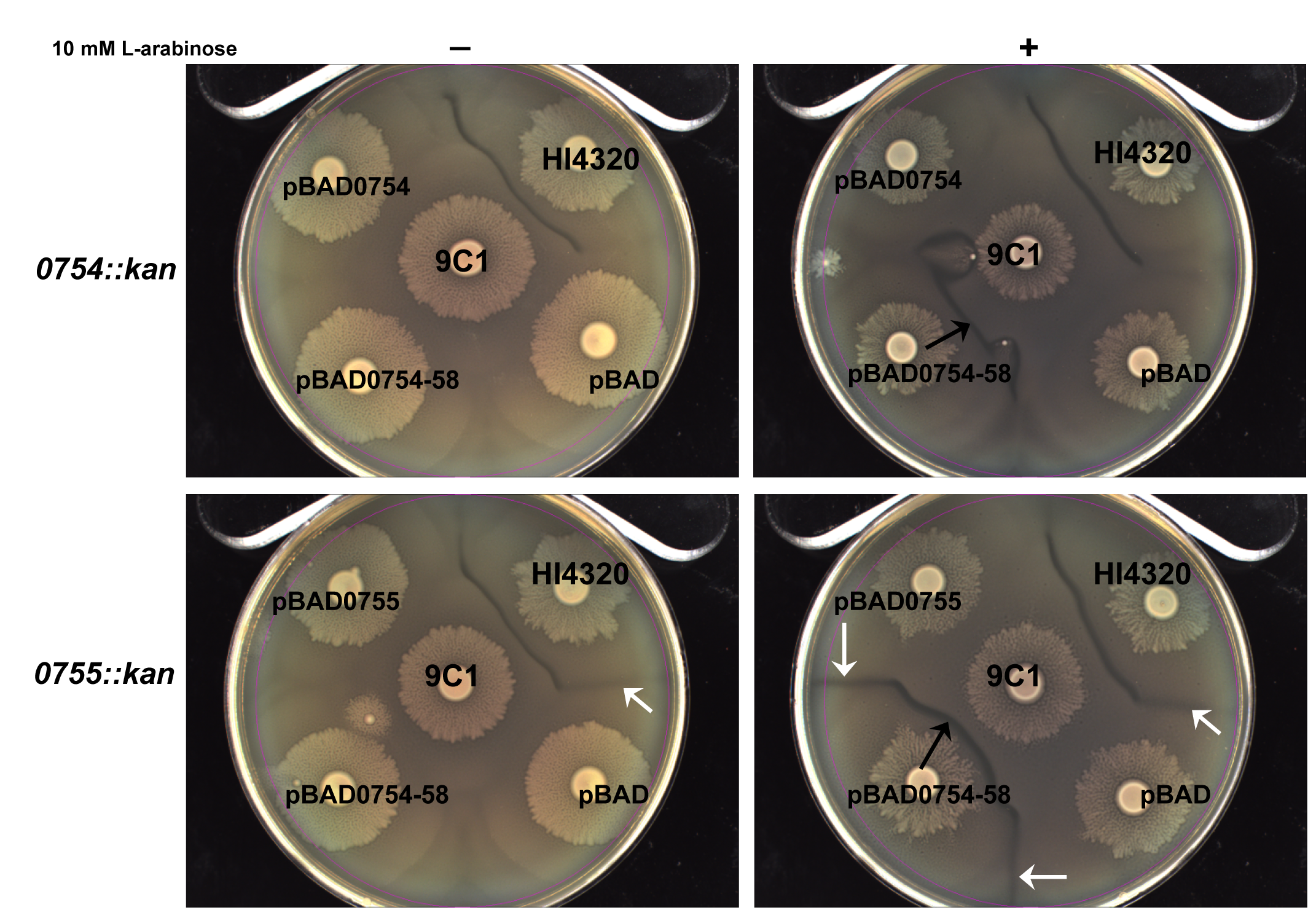

Supplement: Figure S4 — Disruption of PMI0754 or PMI0755 from the primary hcp - vgrG effector operon in P. mirabilis HI4320 abolishes Dienes line formation with 9C1. P. mirabilis mutants in genes PMI0754 (0754::kan) and PMI0755 (0755::kan) containing pBAD empty vector (pBAD), pBAD0754 or pBAD0755, or pBAD0754–0758 inoculated opposing 9C1 onto swarm agar (−) or agar plates containing 10 mM L-arabinose (+). HI4320 containing pBAD was included as a control. 0754::kan and 0755::kan are unable to form a line with 9C1 unless complemented by the disrupted gene plus the remainder of the primary effector operon (black arrows). Note the presence of a partial Dienes line between HI4320 and 0755::kan and between 0755::kan pBAD and induced 0755::kan pBAD0754–58 (white arrows). (TIF) [file ppat.1003608.s004.tif]

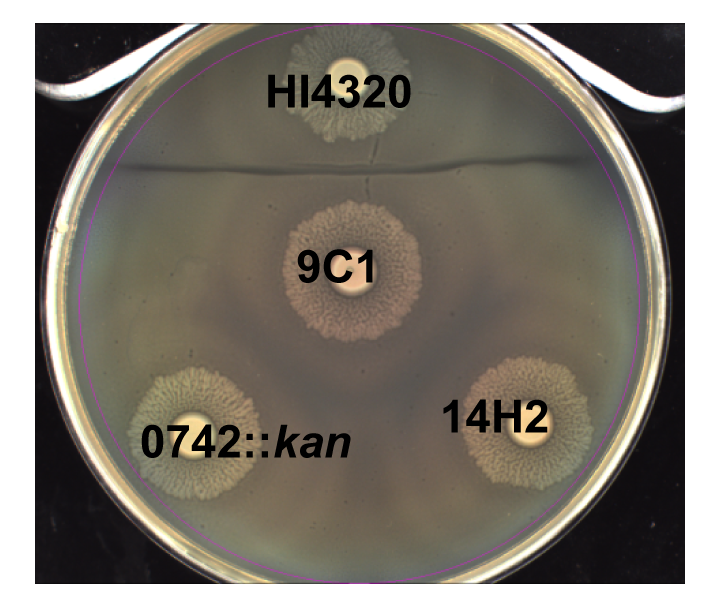

Supplement: Figure S5 — Disruption of PMI0742 abolishes T6SS function. P. mirabilis HI4320 mutant 14H2 has a transposon insertion in the T6SS gene PMI0742 and cannot kill susceptible mutant 9C1. Targeted disruption of PMI0742 (0742::kan) also abolishes the ability of HI4320 to kill and form a Dienes line with mutant 9C1. (TIF) [file ppat.1003608.s005.tif]

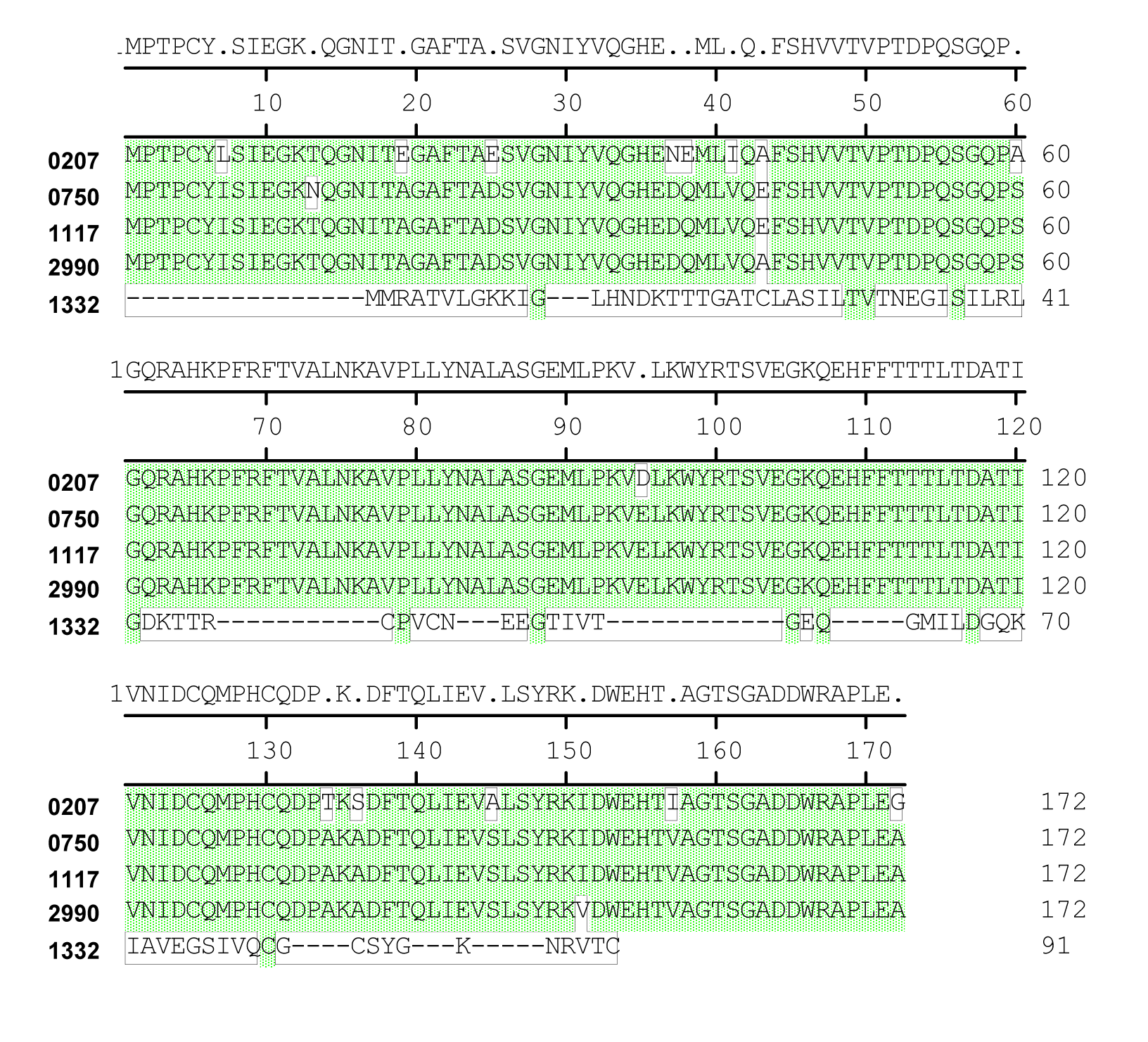

Supplement: Figure S6 — The Hcp amino acid sequences of the hcp-vgrG effector operons in P. mirabilis HI4320 are highly conserved. Alignment of Hcp amino acid sequences encoded by PMI0207, PMI0750, PMI1117, PMI2990, and PMI1332. The Hcp protein sequences of four of the hcp-vgrG effector operons are highly conserved; shaded in green. Boxed amino acids shaded white indicate divergences. PMI1332 is truncated in comparison to the other Hcp proteins by approximately 81 amino acids and as a result has decreased homology to the other Hcp proteins. (TIF) [file ppat.1003608.s006.tif]

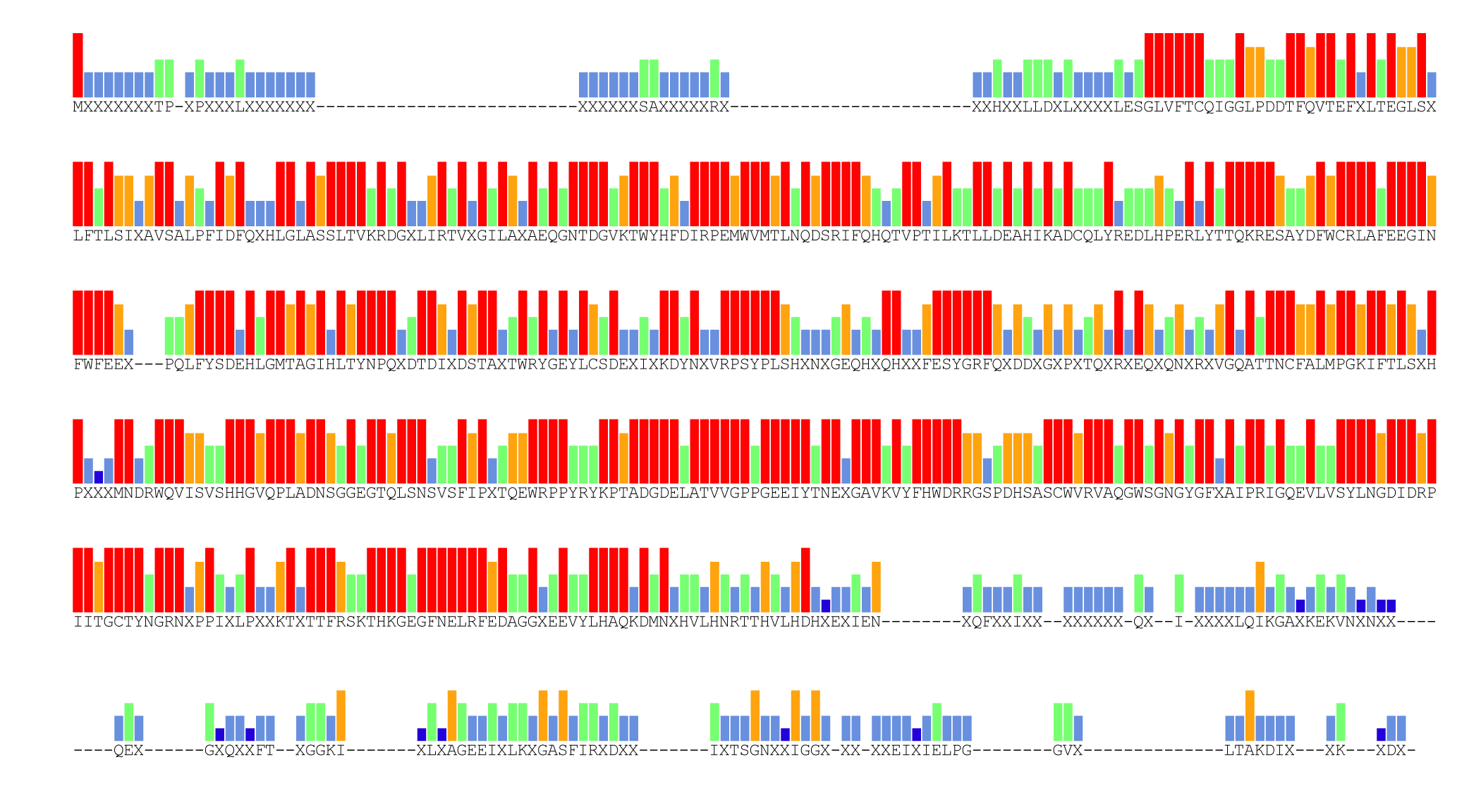

Supplement: Figure S7 — The VgrG amino acid sequences of the hcp-vgrG effector operons in P. mirabilis HI4320 are conserved at the N-terminus and highly variable at the C-terminus. The N-termini of the VgrG homologs encoded by the 5 hcp-vgrG effector operons in HI4320 are highly conserved beginning at the N-termini with decreasing homology beginning approximately 100 residues before the C-termini. Bar colors represent conservation at each position, red; conservation among all 5 VgrG proteins, orange; 4 VgrG proteins share the same amino acid, green; conservation between 3 VgrG proteins, light blue; shared by 2 VgrG and, dark blue; no conservation at that position. (TIF) [file ppat.1003608.s007.tif]

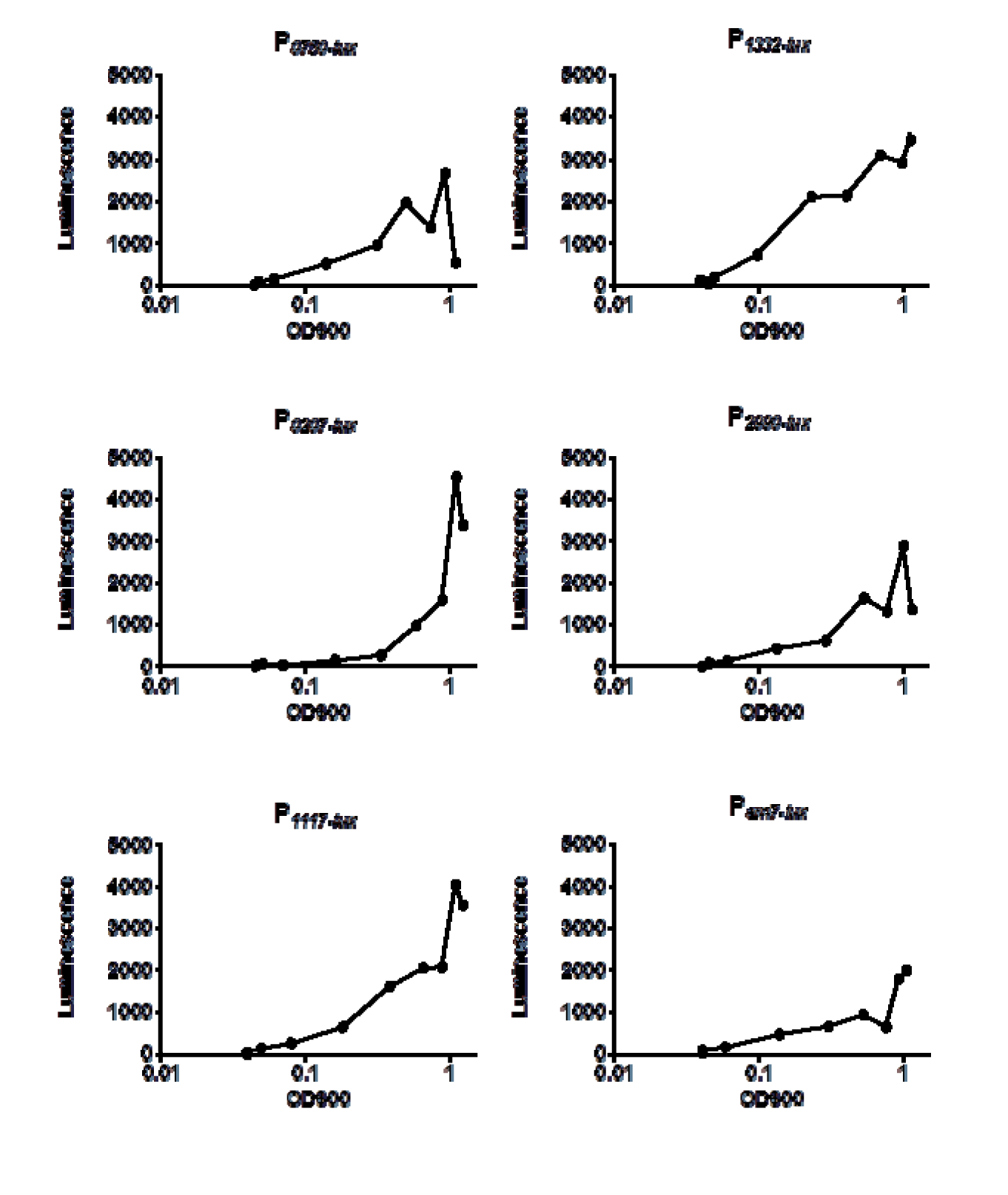

Supplement: Figure S8 — In vitro expression of the hcp-vgrG effector operons in P. mirabilis HI4320 in liquid suspension. P. mirabilis strain HI4320 expressing hcp promoter-luciferase transcriptional fusions were observed over time during growth in LB medium. Luminescence is plotted as a function of cell density (OD600) as measured over time. (TIF) [file ppat.1003608.s008.tif]

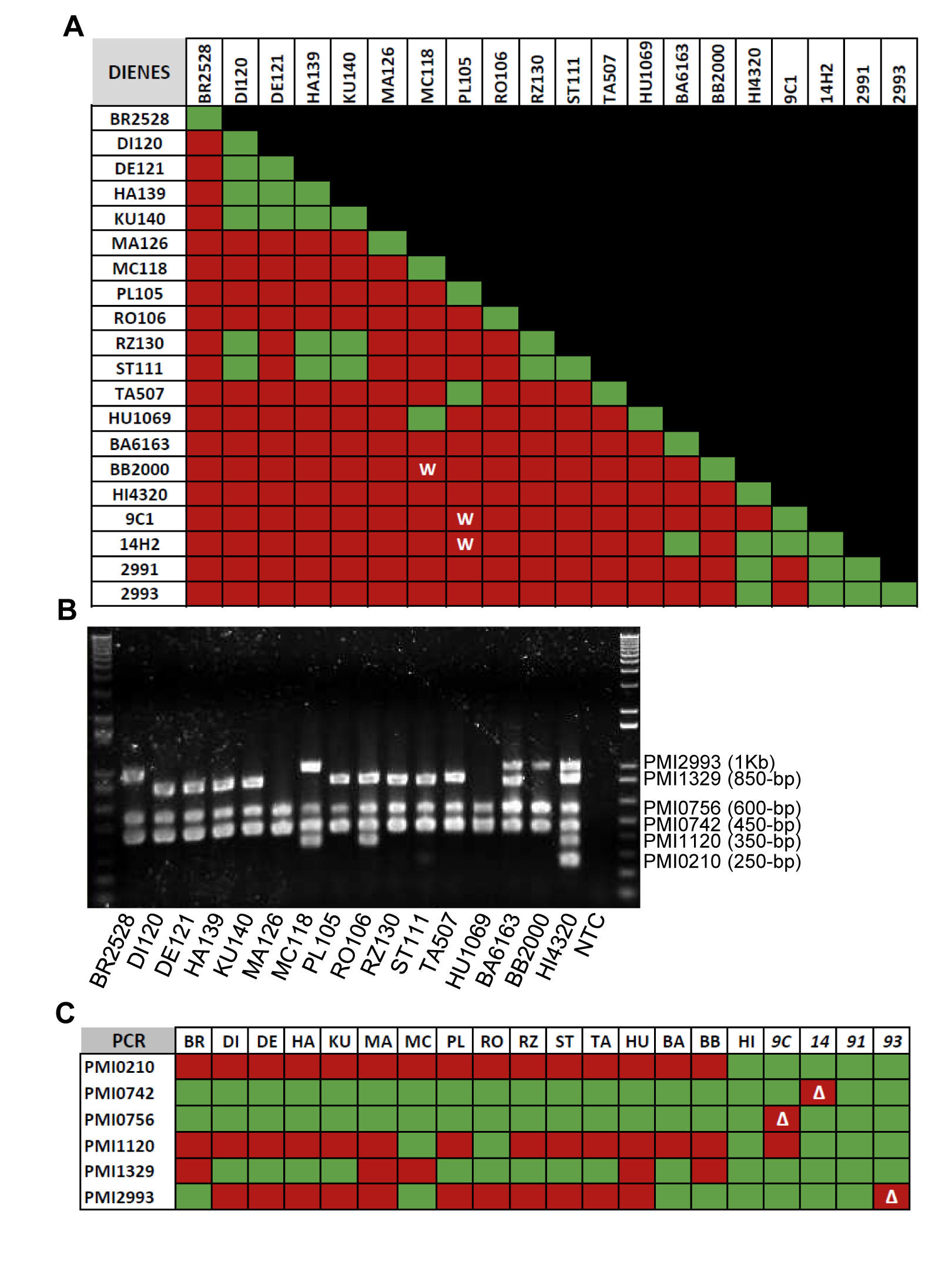

Supplement: Figure S9 — The presence or absence of specific hcp-vgrG effector operons within 16 P. mirabilis clinical isolates does not designate Dienes type. (A) A total of 16 P. mirabilis clinical isolates (BR2528, DI120, DE121, HA139, KU140, MA126, MC118, PL105, RO106, RZ130, ST111, TA507, HU1069, BA6163, BB2000, and HI4320) were examined against each other on swarm agar for formation of a Dienes line. P. mirabilis HI4230 mutant 9C1, T6SS mutant 14H2, and ids mutants 2991 (idsB) and 2993 (idsD) were also tested against the 16 P. mirabilis isolates and each other for a line of demarcation. Red boxes indicate Dienes line formation, green boxes indicate the swarms merged without visible demarcation, and “w” indicates formation of a weak Dienes line. Strains never form a Dienes line with themselves. (B) DNA gel electrophoresis following Multiplex PCR on chromosomal DNA of the 16 P. mirabilis isolates. Primers were designed to amplify one conserved unique gene from each of the 5 hcp-vgrG effector operons and PMI0742 of the T6SS based on the P. mirabilis HI4230 genome. PCR product sizes for PMI0210, PMI1120, PMI0742, PMI0756, PMI1329, PMI2993 are approximately 250-bp, 350-bp, 450-bp, 600-bp, 850-bp, and 1-kb, respectively. (C) Chart showing presence of hcp-vgrG among the P. mirabilis isolates: BR; BR2528, DI; DI120, DE; DE121, HA; HA139, KU; KU140, MA; MA126, MC; MC118, PL; PL105, RO; RO106, RZ; RZ130, ST; ST111, TA; TA507, HU; HU1069, BA; BA6163, and BB; BB2000, HI; HI4320. Green boxes indicate presence of gene (PMI0210, PMI0742, PMI0756, PMI1120, PMI1329, PMI2993) within strain listed and red boxes indicate the gene was undetected within the strain listed. Δ indicates a mutation of the gene in mutant strains; P. mirabilis HI4230 mutant 9C1, T6SS mutant 14H2, and ids mutants PMI2991 and PMI2993. (TIF) [file ppat.1003608.s009.tif]
